# Translating observed household energy behavior to agent-based technology choices in an integrated modeling framework

**DOI:** 10.1016/j.isci.2022.103905

**Published:** 2022-02-11

**Authors:** Oreane.Y. Edelenbosch, Luciana Miu, Julia Sachs, Adam Hawkes, Massimo Tavoni

**Affiliations:** 1 Department of Management and Economics, Politecnico di Milan, Via Lambruschini 4/B, 20156 Milano, MI, Italy; 2 Copernicus Institute of Sustainable Development, Utrecht University, Princetonlaan 8a, 3584 CB, Utrecht, the Netherlands; 3 Department of Chemical Engineering, Imperial College London, London SW7 2AZ, UK; 4 Energy Policy Group, Bucharest 020342 Romania; 5 RFF-CMCC European Institute on Economics and the Environment, Centro Euro-mediterraneo sui Cambiamenti Climatici, Via Bergognone, 34, 20144 Milano, MI, Italy

**Keywords:** Energy sustainability, Energy Resources, Energy systems

## Abstract

Decarbonizing the building sector depends on choices made at the household level, which are heterogeneous. Agent-based models are tools used to describe heterogeneous choices but require data-intensive calibration. This study analyzes a novel, cross-country European household-level survey, including sociodemographic characteristics, energy-saving habits, energy-saving investments, and metered household electricity consumption, to enhance the empirical grounding of an agent-based residential energy choice model. Applying cluster analysis to the data shows that energy consumption is not straightforwardly explained by sociodemographic classes, preferences, or attitudes, but some patterns emerge. Income consistently has the largest effect on demand, dwelling efficiency, and energy-saving investments, and the potential to improve a dwellings' energy use affects the efficiency investments made. Including the various sources of heterogeneity found to characterize the model agents affects the timing and speed of the transition. The results reinforce the need for grounding agent-based models in empirical data, to better understand energy transition dynamics.

## Highlights

• Household survey data used to enhance empirical grounding of agent based model
• New method developed to translate survey questions to model parameters
• Sociodemographics and perspectives do not easily explain consumption behavior
• Income has largest effect on demand, efficiency and energy-saving investments

## Graphical abstract

**Figure:**
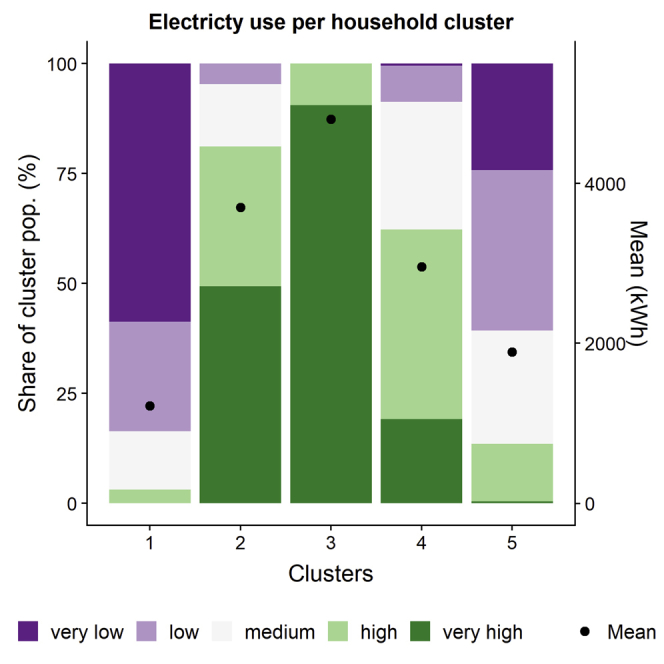

## Introduction

Energy use in residential buildings accounts for approximately 25% of the EU's total final energy consumption (EC, 2018), and has been the focus of numerous emissions reduction programmes, such as the Energy Labeling Directive and the Energy Performance of Buildings Directive. Although the potential for reducing residential energy use is substantial (Berardi, 2017), energy consumption in buildings relies heavily on the resident behaviors, depending on preferences, attitudes, habits, and consumption patterns. Differences in behavior lead to sizable variation in household energy consumption and introduce heterogeneity into decisions on efficient technology adoption and other solutions to achieve low energy and low emissions buildings (Hong, Taylor-Lange, D'Oca, Yan and Corgnati, 2016).

Research efforts to better understand heterogeneous behavior and its drivers are central to successfully implementing residential energy efficiency policies and programs (Lopes et al., 2015). A vast amount of research has examined residential energy-saving behavior, employing a range of disciplinary perspectives ranging from the more economics-focused research frameworks to theories that incorporate psychological, social, and contextual factors (Wilson and Dowlatabadi, 2007). Although the adoption of energy efficient technology boils down to individuals making purchasing choices, it is subject to a complex set of factors, such as social comparisons, being loss or risk averse, or favoring the status quo over change. Investment decisions in residential energy-saving technologies go beyond cost considerations (Klöckner and Nayum, 2017; Hesselink and Chappin, 2019; Wilson et al., 2014), and relate, besides individual motivations, also to their environment (Frederiks et al., 2015; Moglia et al., 2018).

Decision-makers in the energy policy domain are often relying on energy systems modeling, and integrated assessment models (IAMs), that are used to examine long-term pathways of climate change mitigation and conduct global analyses of the energy system (Tavoni et al., 2015). These models represent the different sectors contributing to global carbon emissions and their interactions, to evaluate the cost-effectiveness of climate policy across sectors and regions (van Vuuren et al., 2011). The focus on cost-effectiveness naturally brings an emphasis on costs impacting decision making. For a fair comparison of sectoral efforts, climate policy is commonly represented by a global carbon tax. The models focus on high-level trends (Krey, 2014) that fit the model’s scope. They are as such are less suited to represent the complexities of individual choices, interacting behaviors, and beyond cost considerations, which are shown to be important in the adoption of energy saving technologies (McCollum et al., 2017). The lack of the heterogeneous behavior can lead to several challenges, including inaccurate estimation of demand-side reduction potential (Sovacool et al., 2015), failure to capture interactions such as social influence effects (Pettifor et al., 2017), and difficulties in evaluating the impact of behavioral policies (Mundaca et al., 2010).

Agent-based models (ABMs) have been developed to simulate the complexities of heterogeneous behaviors. ABMs are models composed of agents that interact with their environment and with each other (Squazzoni, 2010). Therefore, the agents are distinct parts of the computer program, and as such, they can capture heterogeneous drivers of decision-making impacted by their individual context (Farmer and Foley, 2009; An, 2012). The idea is that by modeling the micro mechanisms and local processes that underlie the macro outcomes, the dynamics and responses to new situations can be better understood. ABMs are therefore seen as one of the most important innovations in social science in recent decades, allowing researchers to test and analyze social science theories in a tractable manner (Squazzoni, 2010). Given the heterogeneous nature of energy consumers, Rai and Henry (2016) as well as Moglia et al. (2018) make a compelling argument that ABMs should be used to underpin the dynamics and the complexities of energy choices.

Several ABMs that focus on the adoption of energy technologies in the residential sector have evolved. Moglia et al. (2018), for example, apply an ABM model to simulate the adoption of solar hot water systems, Rai and Robinson (2015) describe an ABM that simulates the adoption of residential solar-PV, and Chappin and Afman (2013) use an ABM approach to specifically analyze the adoption of efficient lighting. Some of these studies apply an ABM to a specific country or region level case study, whereas others reflect on a more hypothetical case. For a more general overview, Moglia et al. (2017) provide an in-depth review of the applicability of ABMs to evaluate the technology diffusion in the residential sector. Clearly, empirical validation is not a straightforward task, given the complexities at the micro level (Ghorbani et al., 2015), but is a fundamental step of the modeling process (Boero and Squazzoni, 2005). Moglia et al. (2018) argue that using household surveys as an empirical base to characterize the individual behavior and reasoning is a valid approach. However, the challenge of empirical grounding is perhaps even larger when applying ABM methods to energy systems modeling or IAMs given their broad temporal, geographical, and sectoral scope. There are recent examples of studies with a macroeconomic focus that have taken up the challenge, such as Lamperti et al. (2020); Lamperti et al. (2019), projecting interacting and heterogeneous firms, households/works, energy plants coupled to banks and a government, and impacting climate variables within an IAM framework. Given the large gap between microlevel choice dynamics and global energy trends, there is a need for robust methods that allow modelers to translate empirical data to ABM techniques that can be used in long-term global models.

The aim of the paper is to develop a method to, (a) characterize household energy choices in a way that is appropriate for the scope of a long-term energy model with, (b) an agent based structure that is, (c) based on real household-level energy consumption data combined with survey questions, and (d) use it to understand how characterizing different household groups impacts the projected energy transition. We use a cross-country dataset of household-level survey responses conducted in Italy, the Netherlands, and Switzerland, including sociodemographic characteristics, attitudes, energy-saving habits and energy-saving investments, and metered household electricity consumption. A critical distinction is made between on the one hand the service demand, i.e., the desired services for which energy is required and energy efficiency, i.e., the energy used to fulfill that service depending on, for example, technology adoption. The diversity of questions asked in the survey allows distinguishing between the two.

Using cluster analysis, the dataset is explored to find robust, heterogeneous patterns, and drivers of energy-saving behavior; and crucially, the availability of metered electricity usage data allows one to relate the agent typologies to *actual* energy consumption levels. The research focuses on two main questions: 1) Can the developed cluster analysis method reveal distinct household groups with different energy consuming behavior that provide insights on why households consume energy differently and how they differ in their energy saving investments? and 2) if so, how does translating energy behaviors and characteristics of the identified groups, and the investment drivers, to an agent-based IAM impact the projected technology adoption in the residential energy transition?

The cluster-based analysis results are applied to the Residential Building Simulation Module (RBSM) ABM, part of the MUSE IAM (Sachs et al., 2019). MUSE is an energy systems model with a highly disaggregated and technology rich sector representation. The modular structure of the sectors is brought together in a partial equilibrium through a market clearing algorithm, which balances supply and demand of each energy commodity. The RSBM is one of the sector modules in MUSE, and is a bottom-up, technology-rich model, characterizing 70 different residential energy technologies, based on unit technology cost, efficiencies, lifetime, and emissions. The model uses an ABM method to represent investment decisions via a stepwise decision-making process, including gathering information, evaluating options, and decision-making based on combined objectives. In contrast to other ABMs, the presented approach focuses on a detailed modeling of the investment decision process. The interaction with other agents is represented through the uptake of technology and the observation of the agents of the market share of the different technologies. Overall, the model can be seen as an implementation of an ABM in an IAM framework, representing the agents and interaction structure of the ABM, while allowing users to evaluate whole-system transitions.

In the original top-down formulation, the description of the different agents is informed by the SINUS-Milieu Typology that described population groups by linking demographic backgrounds and social status to people’s values and attitudes across regions (Sachs et al., 2019). These values and attitudes were used to characterize the agents’ objectives and decision making strategy. The empirically-based cluster driven model presented in this paper will be compared to the original top-down SINUS Milieu Typology to understand the impact of the new formulation.

## Results and discussion

### Description of clusters

The clustering was conducted on the continuous demand-related variables (lighting service, appliance service, and efficiency gap). By clustering the demand variables, households that have a similar type of energy demand are grouped together. Then the sociodemographic and environmental preference variables of these clusters are analyzed to understand household characteristics and behavior drivers. Table S1 provides details of the categorization of the variables and the questions asked in the survey on the basis of which these variables were constructed. The categorical values used for the clustering are published, see Zenodo Database: https://doi.org/10.5281/zenodo.5906624.

**Figure 1:**
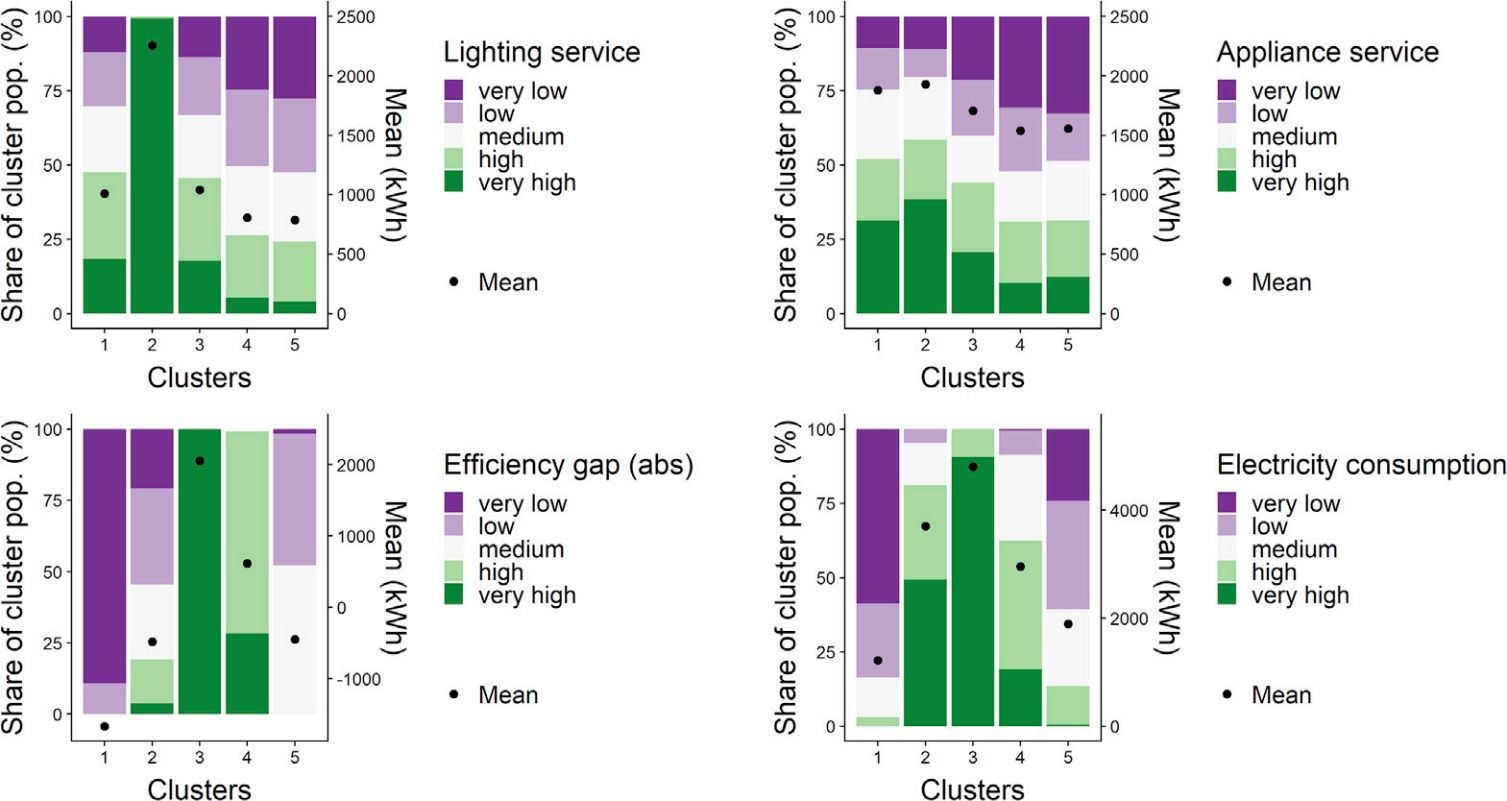
Energy consumption characteristics of the identified clusters The share of the cluster population belonging to an appliance and lighting service, the efficiency gap, and the overall electricity consumption category is depicted. The dot identifies the clusters' mean lighting service, appliance service, efficiency gap, and total electricity consumption expressed in kWh consumed.

**Figure 2:**
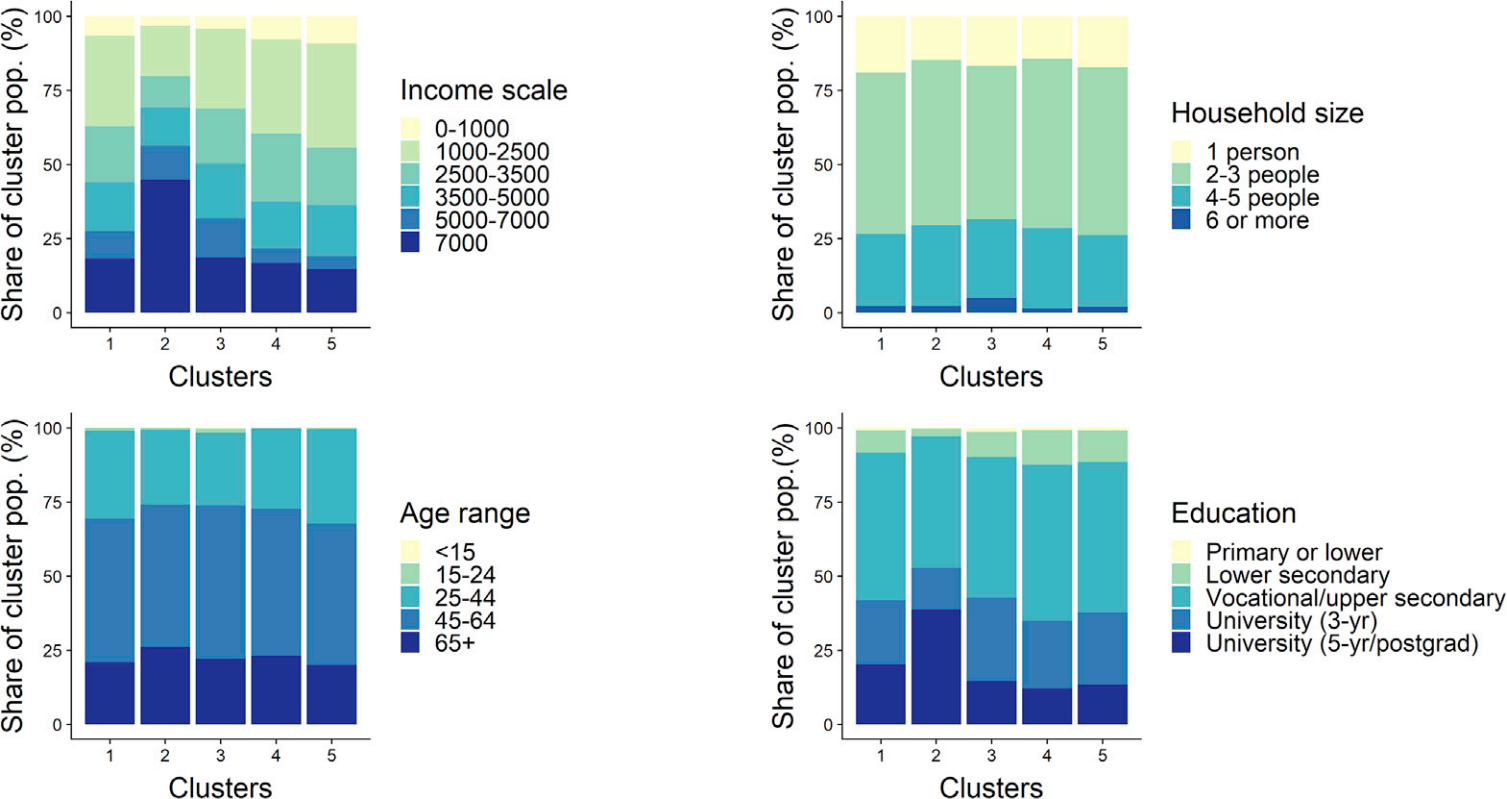
Sociodemographic characteristics of the identified clusters The share of the cluster population belonging to a sociodemographic (income, household size, age range, and education) indicator category is depicted.

**Figure 3:**
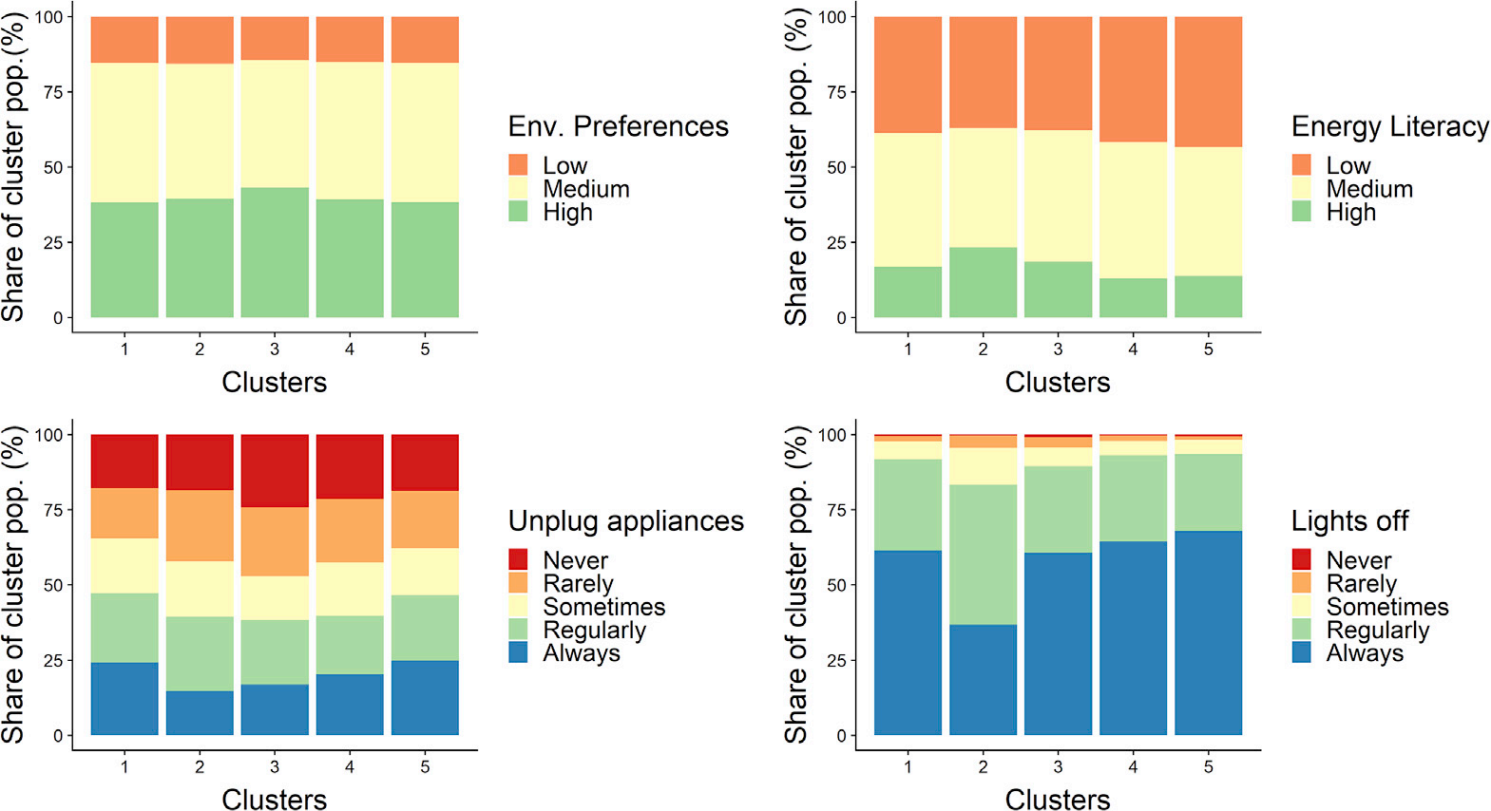
Behavioral characteristics of the identified clusters The share of the cluster population belonging to the environmental preference, energy literacy, and energy efficient behavior category is depicted.

The clustering of the lighting energy service demand, appliance energy service demand, and efficiency gap produced an optimal solution of 10 clusters, containing 4,874 responses. Out of these 10 clusters, five were suitable for further analysis and is presented in Table 1. The Table shows that the distribution of the clusters varied over the countries; however, given the lower response in Switzerland and the Netherlands, a between-country analysis could not produce meaningful results. Therefore in the results that follow we show the total results of all three countries combined. The significance of the difference between the 5 clusters was tested for a set of variables deducted from the survey (see Table 2). Most variables were significantly different between the clusters, apart from household size (although significant at p < 0.1), and the environmental preference index; post hoc tests of difference confirmed this. The differences between clusters were the strongest for the efficiency gap and the relative savings potential. The main characteristics of respondents' grouped in each cluster can be seen in Figures 1, 2, and 3, and are described in more detail below.

**Table 1:** Size of clusters identified in the optimal cluster solution

Cluster	Number of survey responses			
	Italy	Switzerland	Netherlands	Total
1	643 (17%)	125 (23%)	39 (23%)	807 (18%)
2	150 (4%)	147 (27%)	21 (12%)	318 (7%)
3	319 (8%)	86 (16%)	40 (24%)	445 (10%)
4	1,204 (31%)	70 (13%)	34 (20%)	1,308 (29%)
5	1,538 (40%)	116 (21%)	36 (21%)	1,690 (37%)

**Table 2:** Significant differences between the 5 clusters

Variable	Measured Unit	Significant differences between clusters?	Variable	Measured Unit	Significant differences between clusters?
Income class	€/month	Y	Efficiency gap	kWh/yr	Y
Age range	Years	Y	Relative savings potential	%	Y
Education level	Achieved degree^b^	Y	Energy-saving habits: frequency of switching lights off	Scale (1–5)	Y
Household size	Persons/household	N (p = 0.051)^a^	Energy-saving habits: frequency of unplugging appliances	Scale (1–5)^c^	Y
Environmental preference index	Likert scale (1–7)	N (p = 0.46)	Respondents' value of wealth	Likert scale (1–7)	Y
Energy literacy	Scale (low, medium, high^d^)	Y	Dwelling floor area	m^2^	Y
Metered electricity consumption	kWh in 2016	Y	Length of residence in dwelling	Years	Y
Estimated lighting electricity demand	kWh in 2016	Y	Risk preferences	Likert scale (1–7)	Y
Estimated appliance electricity demand	kWh in 2016	Y	Level of energy-saving investments	US$ invested in energy-saving measures in 2016^e^	Y

The variables are categorized in categorical variables constructed based on the questions asked in the PENNY and COBHAM household survey (see for more information the STAR Methods).

a The p values are reported for the Kruskal-Wallis tests that accounted for ties in the data.

b Primary school, lower secondary school, upper secondary school, university, and postgraduate.

c The scale of both energy-saving habits variables corresponds to: one- never; two- rarely; three- sometimes; 4 – most of the time; 5 - always.

d Low energy literacy was defined as the respondent not answering either energy literacy question correctly, medium as answering one of two questions correctly, and high as answering both questions correctly.

e This variable was only available in the COBHAM survey.

Cluster one is *comfortably efficient*, living in very efficient (86%) or efficient (14%) households, despite having a higher appliance service demand than other clusters. Families in this group have already achieved significant savings, and there is little room for further improvement, with annual electricity consumption being on average 58% less than expected demand. Members of this group are relatively young, live in slightly smaller households, and have the lowest residence times. They have good energy-saving habits, particularly being the best at unplugging their appliances, despite having slightly lower environmental preferences.

Cluster two is a *well-off, medium-efficient* group, with a range of household efficiency gaps, the highest lighting and appliance electricity demand of all groups and potential savings of up to 26% (compared to the expected use) in inefficient households, which represent 24% of the group. Members have higher education, strikingly higher income levels, slightly older, more energy literate, and have somewhat worse energy-saving habits than other clusters. They have marginally larger household sizes, live in slightly larger dwellings, and value wealth highly.

Cluster three is an *inefficient, high-consumption* group, and its members live exclusively in very inefficient households, consume the most electricity of all groups, and have potential savings of 64% on average. They have the largest household sizes, are relatively well-educated, and have high environmental preferences, but have the highest proportion of group members who never unplug their appliances to save energy.

Cluster four is an *inefficient low-consumption* group, whose members live in inefficient (57%) and very inefficient (39%) households with average potential energy savings of 28%, have very low lighting and appliance electricity demand, and medium levels of electricity consumption. They live in the smallest households and dwellings of all groups, are relatively uneducated, and have longer residency times than most other groups.

Cluster five, the *resource-constrained and medium-efficient,* is also a low-consumption cluster, where the majority lives in efficient households (92%) or in less efficient households with potential savings of less than 4.5% to consume electricity according to their expected demand. Members of this group have the lowest income, are slightly less energy literate, and live the smallest dwellings of all groups, despite having a similar household size distribution to the comfortably efficient group.

### Efficiency gap

By comparing the sum of the lighting and appliance service demand to the actual metered electricity consumption, two energy efficiency indicators are produced:

• The “efficiency gap:” the difference between metered electricity consumption and the estimated energy consumption based on the service demand (the values can be positive or negative). The values were grouped into five quintiles, ranging from very efficient dwellings (negative efficiency gap, high absolute value) to very inefficient dwellings (positive efficiency gap, high absolute value). Note that the energy efficiency gap is also a concept that has been used within the economics field. It then refers to so-called investment inefficiencies, where despite being profitable, for example, because of imperfect information, firms, or consumers do not invest in efficiency (Allcott and Greenstone, 2012).
• The “relative savings potential:” the proportion of the efficiency gap compared to the appliance and lighting service demand, as an indicator of how close a household is to consuming as much electricity as expected based on its dwelling characteristics. This also gives an indication of the potential energy cost savings. The values were grouped into quintiles, ranging from very high saving potential (i.e., households with the largest positive efficiency gaps, as a proportion of their estimated electricity demand) to very low saving potential (i.e., households with the largest negative efficiency gaps, as a proportion of their estimated electricity demand).

Analyzing the efficiency gap for different groups can reveal how much households are overconsuming or underconsuming relative to their expected electricity demand. For example, a group of households with very low efficiency gaps is already very efficient (e.g., cluster one, which consumes on average 1,546 kWh less than its expected annual electricity demand); a group of households with very high efficiency gaps is very inefficient (e.g., cluster three, which consumes on average 1,737 kWh more than its expected annual electricity demand). It thus has to make substantial savings to reduce its electricity consumption to expected levels. Thus, the efficiency gap describes the “improvement effort” of inefficient households and the “accomplished savings” of efficient households.

On the other hand, analyzing the relative savings potential can reveal how much of a difference reducing this efficiency gap could make to a household: two inefficient households requiring the same improvement effort per kWh may perceive the resulting savings as vastly different if their expected electricity needs are different. For example, despite households in clusters two and five having similar average efficiency gaps (on average −483 and −449 kWh/year, respectively), relative to their electricity demand these gaps are different – lower for cluster two, whose richer, larger households demand more electricity, and higher for cluster five, whose poorer households living in small dwellings demand less.

An analysis was conducted to determine whether the efficiency gaps, in conjunction with the relative savings, had an effect on the energy-saving investments made in each cluster. The analysis could only be performed on four out of the five original clusters (clusters one, three, four, and five), because of the lack of investment data for the well-off medium-efficient group.

**Figure 4:**
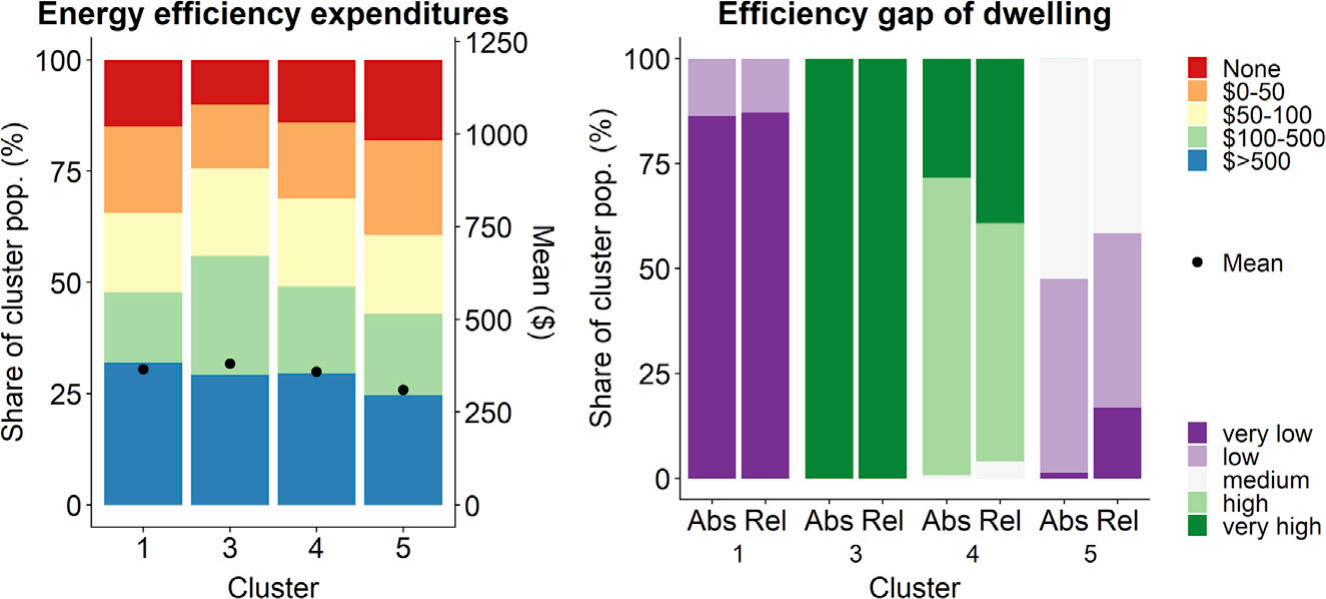
Comparing energy efficiency expenditures to the efficiency gap Left panel depicts the energy efficiency expenditures per cluster. Right figure depicts the relative efficiency gap (rel) and the absolute energy efficiency (abs) for the five clusters.

As shown in Figure 4, the differences in energy-saving investment levels between groups are less noticeable than those in efficiency gaps or relative savings, indicating that investment is affected by more than just these factors. These two variables do affect investment in the two least efficient clusters: clusters three and four, which invested on average $312/year and $299/year, respectively, and around a third of whose members invested at the highest level (over $500/year). However, the distribution of investment levels does not always follow the distribution of efficiency gaps and relative savings (Figure 4). In cluster four, the 52% of respondents who could achieve high relative savings with less effort than those in cluster three, invested about as much as those who needed to put in as much effort as those in cluster three to achieve the same savings. However, respondents who could achieve very high relative savings invested slightly more when the effort required to do so was lower (53% invested over $100/year under high efficiency gaps, as opposed to 43% under very high efficiency gaps). This is because of significant interaction effects between the efficiency gap and relative savings (p < 0.05, ordered Probit regression model): as relative savings increase, so does the efficiency gap, requiring more effort to achieve higher savings potential. Respondents may thus be less sensitive to their achievable relative energy savings than to the required improvement effort. Without these interaction effects, the proportion of investment at higher levels, and likely the average investment in cluster four, would be higher.

Other factors also demonstrate clear effects on respondent investments. In cluster three, larger households (two to three person and four to five person, which make up 80% of the group) are less likely to invest significantly in energy-saving measures than single-person households (p < 0.05 ordered probit regression). In cluster one, which has already accomplished its savings but still invests a very high amount, investments were negatively affected by increasing environmental preferences and positively affected by increasing the energy-saving habit of unplugging appliances (p < 0.05, ordered probit regression). Furthermore, despite still having potential to save energy, cluster five made the least investments in energy-saving measures, and significantly less than other groups. This was because of the significant positive effect of income on investments at the entire survey population: increasing income levels led to an increase in investment, apart from when respondents entered the highest income level (p < 0.05, ordered probit regression model). With cluster five containing the lowest-income respondents overall, an income constraint on this group becomes apparent, in addition to its relatively low potential for savings. This potential income constraint is also manifested when comparing the other clusters: between clusters three and four, the higher-income group is also the higher-investing group. However, income does not seem to constrain investment by respondents in cluster one, a relatively low-income group that invests a high amount regardless.

The cluster analysis of survey data demonstrates several points. Firstly, there is a considerable variation in sociodemographic indicators, energy-saving habits, and energy-saving investments within groups with similar demand and consumption profiles. Secondly, there is heterogeneity in how respondents within the different clusters decide to make energy-saving investments. This confirms previous findings on the range and diversity of factors affecting residential energy demand, consumption, and energy-saving investments (Hesselink and Chappin, 2019; Trotta, 2018; Klöckner and Nayum, 2017; Klöckner & Nayum, 2017, 2017). However, despite this variation, the cluster analysis identified several patterns for the energy-saving investments of different clusters. At the population level, income generally played a significant role in incentivizing investments, in line with a range of existing research (Nair et al., 2010). Additional drivers on energy-saving investments also had clear effects within and between the different groups, indicating that purely income-driven investment decisions are likely to be unrealistic representations of actual uptake of residential energy-saving measures (Wilson et al., 2014). The efficiency gap and relative savings achievable by a household partially drove investment, similar to the findings of Hrovatin and Zorić (2018) and Nakamura (2016), who outline the importance of physical building characteristics and dwelling efficiency in driving energy-saving investments. On the other hand, there were significant interaction effects between the efficiency gap and relative savings of a household. In contrast, income and, within certain clusters, household size and energy-saving behavior, also contribute to investment levels, confirming the complexity of investment decision-making processes and the heterogeneity of drivers for these processes. In the next section, we describe the translation of this heterogeneity in the MUSE RBSM, and discuss the differences in model projections that this may introduce.

### Application of findings to the RBSM ABM

The clusters described above were used to define four groups of agents with different efficiency gaps and drivers for investment in new energy technologies. Given that there was limited data on the investment made by cluster two, this cluster had to be excluded from agent definition. As shown above, energy-saving investment was driven by a variety of factors, including income, which were used to make assumptions about the objectives that drive agents, and the constraints that block them, when they invest in energy-saving measures (Table 3).

• Cluster one had already achieved significant savings, but still invested substantially, driven by good energy-saving habits. We therefore assume that agents associated with cluster one are motivated by the desire to improve their efficiency, and are therefore assigned “efficiency” and “emissions” objectives. The assumption is that efficiency is prioritized over emissions, and therefore the lexicographic decision strategy is applied. Although they invest significantly more than their income would suggest, their relatively low income levels may constrain them from adopting more expensive energy-saving technologies in the future. This behavior is captured through an upper constraint on “capital cost” of an asset to only allow investments in technologies within a certain price range.
• Cluster three had very high potential to improve its efficiency and benefit from large relative savings, and investments were helped by having high income. However, having high improvement potential was not sufficient to incentivize investment from larger households. We, therefore, assume that agents associated with cluster three and living in larger households will be motivated by “efficiency,” “fuel consumption costs,” and “equivalent annual costs” objectives and make the investment decision based on the weighted sum of all three objectives, whereas the rest is motivated by “efficiency” and “equivalent annual costs”. Because people within this cluster show the highest income, the required initial investment is assumed to have only a minor role where the total lifetime cost, EAC, is more likely to be taken into account.
• Cluster four also had high potential to improve its efficiency and benefit from savings, but less so than cluster three. Its overall investment was reduced by the underinvestment from respondents with high efficiency gaps and high relative saving potential, who already had low electricity consumption. Some agents associated with cluster four were therefore assumed to be motivated by the “fuel consumption costs,” “EAC,” and “capital cost” objectives, but only if their electricity consumption was high. The investment decision is based on the weighted sum of all three objectives.
• Cluster five had little potential to improve its efficiency further, having already achieved significant savings. Therefore, most agents were assumed to be driven by the desire to be more efficient, while for those where the potential for savings still exists, by the desire to reduce their energy costs, and are therefore assigned the “fuel consumption” objective. Regardless of their drivers, agents associated with this cluster were strongly constrained by their low income, which caused the lowest investment level of all groups. Thus, an upper constraint on the maximum amount of initial investment is integrated through the epsilon constraint decision strategy.

**Table 3:** Translating the cluster findings intoagent objectives

Cluster	Objectives of agents	Constraints^a^	Openness to new technologies	Decision strategy
Cluster 1 – AGENT 1	Emissions Efficiency	Constrained by capital cost	High – open to invest in new technologies	Lexicographic
Cluster 3 – AGENT 2	Efficiency Fuel consumption costs Equivalized annual cost	Not constrained by capital cost	Low – prefers to invest in mature technologies (10% maturity threshold)	Weighted sum
Cluster 4 – AGENT 3	Fuel consumption costs (partially) Equivalized annual cost Capital cost	Constrained by lower electricity consumption	Neutral – neither takes high risks with new technologies nor prefers to invest in mature ones (5% maturity threshold)	Weighted sum
Cluster 5 – AGENT 4	Efficiency Fuel consumption costs (partially)	Constrained by capital cost	Neutral- neither takes high risks with new technologies nor prefers to invest in mature ones (5% maturity threshold)	Epsilon constraint

Note that cluster two was not included in the modeling because of limited investment data. The formulation of the different decision strategies can be found in the STAR Methods.

a Note that capital cost constraint is relative to income; therefore, cluster five which has a lower income is more limited in its choice than cluster one.

The survey data collected on risk preferences and energy literacy were also used as a proxy for agent openness to new technologies for each cluster, which defined their rules for searching for new energy technologies and the desired maturity level when deciding to make an energy-saving investment (Table 3). Risk preference refers to the respondent’s attitudes toward risk, being risk prone, or adverse, whereas energy literacy refers to respondent knowledge toward energy costs and savings.

These assumptions on objectives and constraints were used to define the four agents within the RBSM for the EU-18 region, and project the uptake of heating and lighting technologies in this region, up to the year 2050. The key changes to the model are the share of the population represented by an agent, the demand for energy service, the technology maturity threshold, and constraints on the budget. The assumption here is that the energy consumption patterns seen for lighting and appliances will also be reflected in other end-uses of energy for residential buildings.

We also examined the effect of adding (1) within-cluster variation in service demand, by creating “clones” for each agent, with the same sociodemographic profile but different demand levels; and (2) stochastics around the parameters of agents' decision heuristics (e.g., weights assigned to the agents' objectives when deciding whether or not to invest in a technology) to capture the heterogeneity in decision making of an agent. Five different electricity demand levels are modeled by using a scaling factor based on the electricity consumed in each specific level, compared to the total average energy consumption across all clusters. To capture the within-cluster variation, each of the agents ‘clones’ a certain share of the in-cluster population belonging to one demand level. The stochastics are implemented by multiplying the decision heuristic with a scaler drawn from a normal distribution with mean one and variance of 20%. Every time the decision process is carried out, a random value from this distribution is taken and multiplied to the decision heuristic.

**Figure 5:**
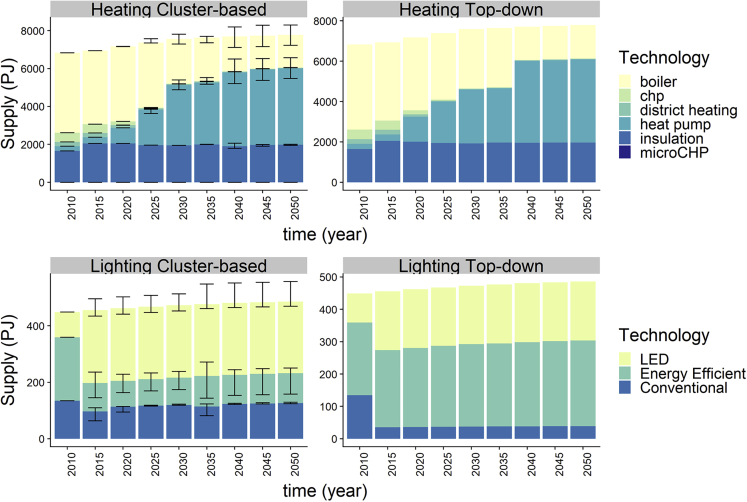
Projected lighting and heating technology penetration Left panels show the cluster-based approach including different service levels. Right panels show a top-down driven approach. The cluster results include the spread in technology uptake over the 100 runs, indicated by the error bar.

Figure 5 shows that the projected uptake of heating and lighting technologies changes based on the agents derived from the cluster analysis versus the original top-down approach. There is an increased uptake of LED lighting in the cluster based model, compared to the original top down formulation, where efficient lighting bulbs dominate. The two approaches show a similar technological landscape for heating in 2050, where heat pumps will be increasingly adopted will and replace conventional boilers in response to the increased energy cost because of the carbon tax implementation. However the transition dynamics are somewhat different. In the cluster-based model, initially heat pumps have a smaller share, but then experience a more distinct transition phase with a rapid uptake in the period 2025–2035. The original model, in contrast, shows a more gradual transition. We examine the differences between the two models by using an ANOVA test and find the two models to be significantly different for lighting technology, but not for the heating technologies. Given that the residuals are not normally distributed, the nonparametric Kruskal-Wallis test is used, which is less strong but valid for non-normal data. Both time and technology use have a significant effect showing that the rate of adoption is a key model characteristic; however, for lighting, only technology use is significantly different (see for more details the Tables S5 and S6.

**Figure 6:**
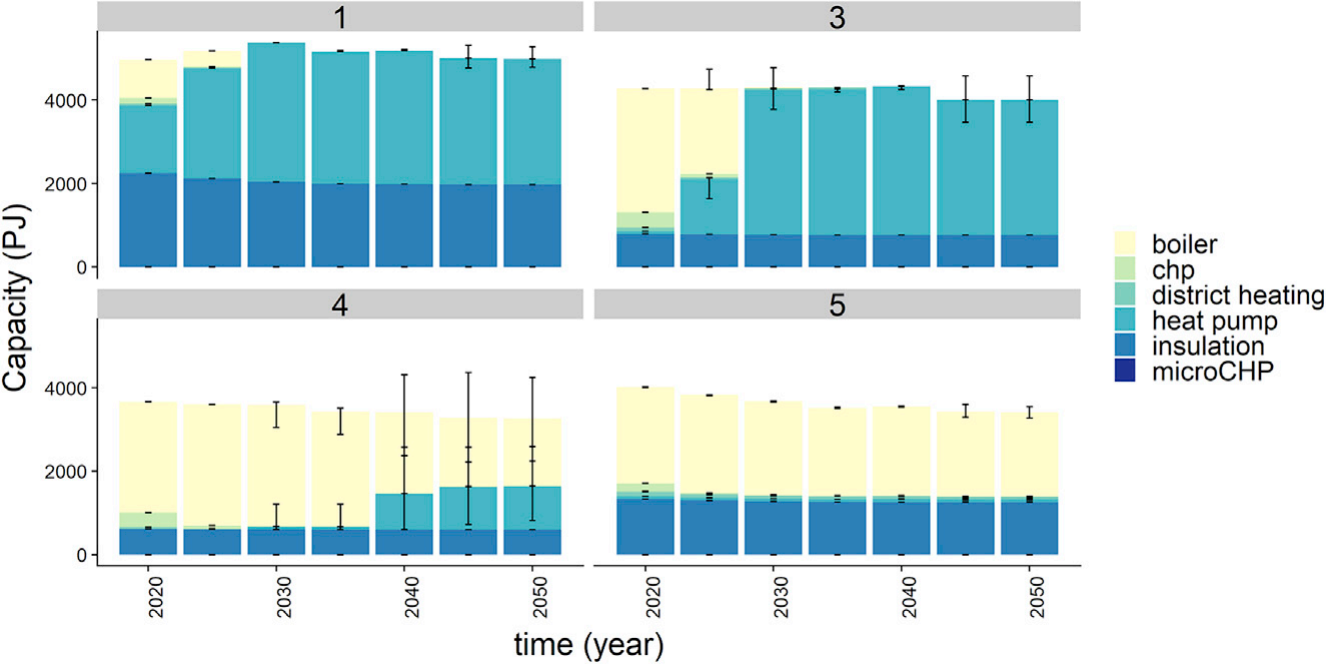
Heating technology penetration in the cluster-based approach The model is run with different service levels for the clusters 1, 3, four, and five impacting the results. The figure shows the spread in technology uptake over the 100 runs, indicated by the error bar.

These different diffusion patterns are the result of assumptions on the sociodemographic characteristics of an agent and consequently the share of the population presented by each agent, its financial limitations, and openness to new technologies. For example, in the original top-down approach, the assumption that all high-income, well-educated agents within a certain age group tend to adopt energy-efficient technologies, and this leads to an uptake of heat pumps in all high-income groups, and therefore a more gradual diffusion to other groups over time. In the cluster-driven model, the heterogeneity in investment drivers across agents (also within high income groups), affected by the role of efficiency gap and relative savings, means that uptake of heat pumps will start earlier and has the potential to then grow quickly (see also Figure 6).

Figure 6 shows how the installed heating technology capacity differs for the four clusters. Cluster one is the first to move to heat pumps as they are open for new technologies and are attracted by efficiency improvements. Cluster three, not being very prone to new technologies, initially showed lower heat pump adoption rates compared to cluster one. As the technology becomes more mature and attractive, from a cost perspective, given their fuel cost and equalized annual cost objectives, a full transition is seen. Importantly, cluster three is not constrained by capital costs. This seems to be a large hurdle for cluster four and even more for cluster five. The large uncertainty surrounding the heat pump adoption of cluster four after 2040 suggests that targeting this group with investment subsidies targeting their capital cost objective might tip them over the edge. Cluster five on the other hand, despite being attracted by efficiency gains are less likely to transition to heat pumps because of the high investment costs. Moreover, given the lower service demand of cluster four and five, the relative potential savings of installing a heat pump is lower. Note that the capacity installed for clusters one and three is relatively high compared to four and five, despite representing a smaller share of the population because of their high service demand.

A similar figure showing the lighting technology choices per cluster is presented in the Figure S10. It can be seen that not only clusters one and three shift to LEDs, although cluster four has partly adopted LED lighting already in 2020. This choice is motivated by their objective to reduce fuel consumption cost and EAC, although the capital costs of LEDs are not limited by their capital cost objective. Interestingly, the share of conventional lighting in the new formulation is larger as well, because of the mixed use of conventional and efficient lighting by cluster five, a group that takes up a relatively large share of the population. Compared to the original formulation, the groups that are inclined to adopt efficient technologies are larger; however, this effect is more visible in the lighting technology choices as they are less impacted by high capital costs.

We tested how including within-cluster variation in service demand impacts the technology adoption results (see supplemental information Figure S7). The results show a higher number of extreme scenarios, identified as outliers, during the transition phase around 2030 and a higher variation in the total uptake toward 2050. Low-carbon technologies may become more competitive around 2030, because of the increasing carbon tax, and a small change in the investment heuristics that leads to the adoption of different technologies and thus different transition patterns. Owing to the introduction of varied service levels, agents with less demand will be less incentivized to adopt high efficient technologies quickly, following the lower energy saving potential. This is obvious for the uptake of lighting technologies, where LED lighting is taken up more rapidly in the varied service level scenario.

### Key outcomes

Agent based models (ABMs) are powerful tools for representing heterogeneity but are challenged by the need for empirical grounding of agent definitions and behavioral parameters. This challenge is even more prominent in the context of long-term energy projections, such as those represented by IAMs. In this study, we use a cross-country survey to identify patterns and drivers of energy-saving investments. By estimating household-level electricity demand and comparing it to metered electricity consumption, we defined measures of dwelling energy efficiency. We used these to conduct a cluster analysis and partition the large survey population into groups with characteristic demand and consumption profiles. These groups formed the basis for more in-depth investigation into sociodemographical characteristics, dwelling improvement potentials, and investment behaviors.

The cluster analysis showed significant variation in energy consumption and dwelling efficiency between groups, but much less in sociodemographic characteristics. This highlights the challenges of collapsing energy use patterns to sociodemographic classes. There are, however, revealing patterns both between and within the clusters. Income, consistently, is the biggest driver that affects demand, dwelling efficiency, and energy-saving investments. Dwelling improvement potential also plays an important role, incentivizing energy efficiency investments in households where they might have a significant impact. However, there is interference from other sociodemographic and psychosocial characteristics, such as household size, age, specific groups, environmental preferences, and energy-saving habits. By translating these drivers and user characteristics into ABM objectives and agent typologies, respectively, we have developed a more empirically grounded MUSE RBSM. The individual rich narratives described in this paper based on the cluster analysis are possibly informative for more complex ABM approaches; however, our analysis focused on a simple and transparent approach capturing heterogeneous investment behavior – appropriate for the wide scope of an IAM. As undertaken here by including stochastics in several model parameterizations, a systematic treatment of uncertainty can better reflect the variety and diversity in the observed energy choices.

The results show that cluster-based modeling of investor agents leads to different uptake of heating and lighting technologies, in terms of speed and timing of the projected technology transition, and openness to adopt more efficient technologies. By adding uncertainty around the parameters of agent decision heuristics, further variation in the speed of the uptake of heating and lighting technologies appears. The modeling results identify the group-specific barriers and enabling factors to adopt efficient technologies. Because of investment made by the *comfortable efficient* cluster one, the initial hurdle to new technologies of the *well-off medium efficient* cluster two is overcome. Both relatively affluent groups with high service demand, not constrained by capital costs and responding to the implemented carbon tax, transition to efficient technologies by 2030. Although these groups are relatively small, they contribute to a significant part of the energy consumed. The *inefficient high consumption* group is constrained by capital cost but also sees high potential savings. This group, therefore, shows the largest uncertainty in adoption rates, and is likely sensitive to additional policy measures, whereas the *resource constrained inefficient* group is less likely to switch to efficient technologies.

Capturing heterogeneity in long-term models is complex and time-consuming because of the large data requirements. However, in the current age of big data collection, the potential for characterizing household energy consumption combined with questionnaires to clarify household motivations, as was undertaken in this study, is promising. This study demonstrates a methodological approach to link empirical data to an ABM, including agent interactions and heterogeneous investment decision-making, within an IAM framework. The results show that including this heterogeneity in long-term projections can affect transition dynamics, particularly in terms of timing, which is surrounded by large uncertainties and can be impacted by policy. Recognizing that this research is merely a first step in that direction, we encourage future research to continue to use ABM techniques to explore and better understand the fundamentals of long-term transition dynamics.

### Limitations of the study

The more than 6,000 surveyed households across three European countries allowed distilling distinct energy consumption patterns. However, surveying the households over a longer period of time and including more regional differentiation in the dataset, could potentially create more accurate and reliable results that consider different contexts. Many households in the Netherlands and Switzerland that were part of this study shared their electricity consumption data, but did not fill in the questionnaire. The respondents sample size was therefore too small to consider cross country differences. Underpinning long-term dynamics and cultural differences could improve the translation to the IAM modeling and also possibly improve the understanding on how changing the context could impact the household energy choices. Moreover, several other factors could be incorporated for improved analysis of investment behavior: perceived consumer effectiveness – particularly in the case of studies based on self-reported investment (Armitage and Conner, 2001), actual energy savings investment data, and contextual influences such as neighbourhood characteristics, home tenure, and property characteristics (Wilson et al., 2014).

This study has collected metered electricity consumption; therefore, neglecting the consumption of other energy carriers. As a result, the cluster analysis focused on the use of lighting and appliances and for equal comparison had to exclude those households that used electricity for other purposes such as cooking. The patterns distilled in the lighting and appliances data were translated to all residential energy functions in the RBSM model, whereas ideally specific energy consumption data on space heating and cooling would be included in that translation.

The paper shows, based on the concepts of energy service and energy efficiency gaps, a methodological procedure that can be applied to household survey data to distinguish household clusters with specific behavioral patterns. The paper also demonstrates through the model application how the identified clusters can then be translated to model parameters, leading to specific transition dynamics in the projections where the identified clusters have a distinct role to play. Understanding how policy measures can be directed and possibly impact the cluster behavior and transition dynamics, is beyond the scope of this paper, also because of the data limitations, but is clearly an important next step.

## STAR★Methods

### Key resources table

**Table:** 

REAGENT or RESOURCE	SOURCE	IDENTIFIER
**Deposited data**		

Statistics on Income and Living Conditions	European Union Statistics on Income and Living Conditions	https://doi.org/10.2907/EUSILC2004-2018V.1
IEA Statistics	International Energy Agency Statistics	https://doi.org/10.1787/9789264095243-en
EEA Energy consumption per dwelling	European Environment Agency	https://www.eea.europa.eu/data-and-maps/daviz/energy-consumption-by-end-uses-3
Sinus Milieus data	Sinus-Sociovision	http://www.sinus-institut.de/sinus-loesungen/sinus-meta-milieus-weltweit/
Technology roadmap energy-efficient buildings: heating and cooling equipment,	International Energy Agency	https://www.iea.org/reports/technology-roadmap-energy-efficient-buildings-heating-and-cooling-equipment
Categorical variables based on cross country household survey on energy consumption	PENNY and Cobham survey	https://doi.org/10.5281/zenodo.5906624

**Software and algorithms**		

Python (MUSE model)	Python Language Reference	https://www.python.org/
STATA 15® (cluster analysis)	*Stata Statistical Software: Release 15*. College Station, TX: StataCorp LLC.	https://www.stata.com/

### Resource availability

#### Lead contact

Further information and requests for resources should be directed to and will be fulfilled by the lead contact, Oreane Y. Edelenbosch (o.y.edelenbosch@uu.nl)

#### Materials availability

This study did not generate new unique reagents

### Method details

The data used in this study was collected via two large-scale surveys conducted in Italy, Switzerland and the Netherlands. A total of 6,138 responses were recorded, containing information on socio-demographic and socio-psychological characteristics, dwelling and household characteristics, technologies and energy services used, and their metered electricity consumption. There were a large number of missing responses for metered electricity consumption in the Netherlands, leading to an under-representation of data from this country

The survey responses were used to construct newly defined energy efficiency indicators, and energy service indicators. This allows two distinct factors to be separated: service consumption, and energy efficiency relative to the demanded service. Firstly, dwelling characteristics and survey responses related to energy services (e.g. floorspace, ownership of specific appliances and number of lightbulbs), were regressed to the collected metered electricity data. For each household, this allowed us to calculate the expected lighting and appliance electricity demand based on the level service that the household demanded. In this paper this is referred to as the lighting and appliance service demand indicators. The idea is that a larger house, or a house with more appliances for example is expected to use more electricity. Relative to this expected electricity demand energy efficiency can be calculated.

The survey responses were clustered based on the lighting service demand, appliance service demand and the efficiency gap (k-means clustering with Jaccard dissimilarity measure). Because the sum of these three clustering variables is equal to the metered electricity consumption, the metered electricity data itself is not added to the clustering variables. Those household with electric heating or cooking (which were a small number of respondent households) were taken out of the data sample. The cluster outcomes were then analysed for within- and between-group differences in socio-demographic and socio-psychological characteristics, energy literacy, metered electricity consumption, energy-saving habits and energy-saving investments, and environmental preferences. The environmental preferences index was constructed as a simple aggregate of respondent scoring of questions on environmental value, morality, identity and injunctive norms. The social-demographic factors are important drivers of change in the long-term. The variables which showed the greatest between-cluster differences were also fitted to regression models, to determine whether they were significantly affected by other variables. Table S1 provides an overview of all variables analysed, and how they were constructed based on the survey questions.

The resulting clusters were used as a basis for defining and characterizing agents in the RBSM, and projecting the uptake of residential technologies in the EU-18 residential sector, to the year 2050. The age, income and household size distributions within each cluster were linked with data from the European Union Statistics on Income and Living Conditions (EU-SILC, 2019), to determine the overall share of the European population represented by each agent. The EU18 is treated as one region in the MUSE model and as such cultural differences between countries are not explicitly considered. EU-18 refers to EU-18: Austria, Belgium, Czech Republic, France, Germany, Greece, Hungary, Ireland, Italy, Luxembourg, Netherlands, Poland, Portugal, Slovak Republic, Spain, UK, Slovenia, Estonia (IEA, 2017). However, the economic and demographic developments can lead to larger or smaller shares of identified clusters over time.

In the new RBSM model the agents investment decisions are based on the relationships between household characteristics and different investment behaviour identified in the cluster analysis. The model has a relatively high level of technology detail, considers agent-specific objectives and decision strategies. The focus on investment behaviour in particular follows the interest to analyse the adoption of energy efficient technologies in response to climate policy, which, in line with the common IAM approach to climate policy, is approximated through a carbon tax. For our analysis the carbon tax pathway developed for the commonly referenced Energy Modelling Forum study, under the assumption of full technology availability, and corresponding to radiative forcing in line with atmospheric concentrations of 450 and 550 ppm CO_2_ equivalent (CO_2_eq) is used (Kriegler et al., 2014). This study does not directly account for the changing carbon intensity of electricity, but rather considers the influence of the carbon price on the electricity price as a proxy.

The narratives derived and described based on the cluster analysis are translated to specific model parameters. In RBSM the investment decision algorithm is based on the assumption that individuals have the ability to gather information on the available technologies to provide the service they desire and based on their own heuristics and decision making process. This is translated for each model agent in to certain attributes. The most important attributes are: their objective in purchasing a technology, the so-called search space, which are the technologies that they consider to purchase, and the decision strategy which is the method used to combine objectives to make a decision. The drivers of energy-saving investments identified in the survey data were used to make assumptions about what objectives agents would seek to fulfil as they make decisions on investing in energy-saving measures, along with other agent attributes. The main investment objectives used in the RBSM ABM are:

• Capital cost – agents seek to invest in technologies of a certain capital cost based on their income constraints and risk preferences;
• Equivalent annual cost (EAC). The equivalent annual cost of owning, operating (incl fuel costs), and maintaining an asset over its entire life. - agents will seek to adjust their EAC based on their income constraints and savings potential;
• Fuel consumption cost – agents will seek to reduce their fuel consumption cost based on their savings potential;
• Efficiency – agents will seek to adjust the efficiency of their energy use for non-economic reasons
• Emissions – agents will seek to adjust their emissions levels based on their preferences such as environmental awareness.

The interaction between agents is modelled through the search space of the agent, which are technologies that the agent considers to purchase. Depending on agent openness to new technologies, and the maturity of a technology, a technology will be considered in the search space of the agent. Thus, if a technology has been purchased more frequently by certain agents, which could for example also be due to alternating objectives, it will be more likely to enter the search space of other agents. If an agent is more concerned by the behaviour of others, it will have a high technology maturity threshold, and will adopt only after a sizeable share of a technology is in the market. Sachs et al. (2019) provides a more detailed description of the RBSM model which is summarized below.

Each cluster is assigned a percentage of the population, based on income, household size and age from the European Union Statistics on Income and Living Conditions (EU-SILC, 2019). This resulted in to respectively a population share of 18%, 12%, 32%, 39% for cluster 1, 3, 4 and 5. Note that cluster 2 was excluded. This is further broken down into demand levels within the cluster. The percentage of people represented by an agent is split into clones based on the percentage of people assigned to a service demand level. The demand to be served by an agent is then adopted based on the service level through scaling. Agents have to invest in technologies to meet the projected demand assigned to them. Depending on the technology share an agent currently has the demand is split to be met by all technologies based on their capacity. The model was calibrated using the 2010 International Energy Agency Statistics (IEA 2010) provided for the residential sector, combined with European end use level data from the European Environment Agency (EEA 2019).

We compare the outcomes of the new empirically based cluster-driven model to the original, top-down SINUS Milieu Typology agent parameterization (Sinus-Sociovision, 2017). SINUS milieus provides a qualitative description of nine different groups based on peoples demographic backgrounds, occupation, education, values, attitudes and expenditures. The combination of the social status and the values are mapped to 7 groups for the EU (see group description Figure S5). The demographic background described by the SINUS-Milieu Typology is used to map the groups and distill the groups sizes, based on for example survey data of household representative persons (HRP) (Chamberlain 2015), that contain information per household on income, age, and occupation. The described values and attitudes were related to objectives and decision strategies in the RSBM model. The details of this mapping can be found in Sachs et al.(2019). A key improvement in the new cluster-based approach is that the agent definition is based on actual household and energy data, where micro level explanatory factors of heterogenous investment decisions and energy service demand are distilled. Each agent was duplicated to indicate whether their investment was in a new residential building or an existing one (retrofit). The cluster-driven model simulates 5 agent clones for the 5 sub-ranges of estimated electricity demand in each cluster.

#### Energy service indicators

In demand-side mitigation a distinction can be made between *service demand reduction* and *energy efficiency* improvements to reduce energy demand (Fell 2017). Changes in energy service could be seen as lifestyle change, while changes in energy efficiency related to technology choices (Creutzig et al., 2018). To distinguish between these two factors contributing to energy consumption, for the clustering three energy demand related indicators were used (lighting service, appliance service and absolute efficiency gap) that were constructed based on the survey response and measured electricity consumption. Here we discuss how the service indicators were constructed.

The data analysis focuses on the energy services that require electricity, namely appliances and lighting. This allows us to compare the level of service of these end uses to the electricity consumption data collected. Those households that had electric heating were taken out of the data sample (in the PENNY survey this was <5%). To differentiate between electricity consumption for lighting and for appliances, the lighting electricity demand is estimated, based on survey responses on floorspace, the number of lightbulbs, and the share of efficient lighting in the households. This is subtracted from the metered electricity data to estimate the appliance electricity demand.

Key drivers of expected appliance electricity demand, that relate to service, are number of appliances and appliance type (Cabeza et al., 2014). Both in the PENNY and Cobham survey questions are asked on the number of appliances, type of appliance (e.g. dishwasher, tv, washing machine, fridge) and appliance age, and efficient use (e.g. switching off appliances, hanging clothes). In the PENNY survey there are also questions on the frequency of appliance use. The type of appliance and the efficient use questions differ per survey. Our approach is first to analyse for each survey the significance of each component through linear regression analysis in relation to the electricity consumption (see Tables S4 and S5, column p value).

The results show that appliance ownership, and specifically having an additional fridge, freezer, air conditioning, dishwasher, dryer, washing machine, tv, pc or luxury appliances, has a significant effect on electricity consumption, indicated by a p value lower than 0.01. Interestingly, the estimate value (see Tables S4 and S5, column estimate) of the ownership of these specific appliances, calculated by the regression analysis performed against the annual metered consumption data, matches approximately the annual electricity consumption of the individual appliance. For example, the estimated value of a washing machine is approximately 390 kWh/year in both the PENNY and Cobham results, while the dishwasher estimates range between 163-365 kWh/year. Appliances that most people have, such as a fridge (see Table S3), are less likely to be a distinguishing factor between household consumption because they are so common and therefore do not show to have a significant effect on household electricity consumption. The electricity consumption resulting from the use of the commonly used appliances is incorporated in the intercept, representing a base level consumption, and possibly also partly in the estimates of the other appliances. The regression results also show that the estimated lighting electricity demand has a significant impact, with a weight of 1.2 in Cobham and 2.6 in PENNY, showing that the calculated lighting is possibly slightly underestimated in PENNY.

Finally, combining both surveys and regressing only those household characteristics (i.e. ownership of specific appliances and lighting demand) that had a significant impact, we compute for each household the approximate electricity consumption. This approximate electricity consumption is what we call the energy service index, which can be seen as the expected electricity consumption, given the consumption patterns distilled in the data sample, for the level of service demanded in the specific household. This value is compared to the actual energy consumption. The difference is used as the absolute energy efficiency gap indicator.

#### Cluster analysis

The clusters used in this analysis were produced in STATA 15® using K-means clustering. We opted to run the clustering on the dummies (indicator levels) of the clustering variables enumerated above, as this was overall the most efficient clustering method available for these types of variables. The Jaccard similarity measure was used to produce cluster solutions of 1–20 clusters, which were then analysed using analysis of variance (ANOVA) to identify the optimal solution. The within-sum of squares (WSS), its logarithm (log(WSS)), eta-squared (η^2^, similar to the R^2^ parameter) and the proportional reduction in error (PRE) were used as parameters to identify possible optimal cluster solutions. A well-performing cluster solution has a low WSS and log(WSS), a high eta-squared and a high (positive) PRE (Makles 2012).

An additional Ward’s linkage clustering, using the same similarity measure and variables, was run to check the cluster stopping rules for additional information about an optimal cluster solution. While Ward’s linkage clustering is normally run to produce a dendrogram, in this case the data had too many ties to visualize a dendrogram. The stopping rules used for optimal solution identification were the Calinski-Harabasz and Duda-Hart stopping rules. The parameters produced by the stopping rules (Calinski-Harabasz pseudo-F and Duda-Hart Je(2)/Je(1), and Duda-Hart pseudo-T) characteristically have large values and a small value, respectively, for cluster solutions that perform well (Halpin 2016).

Figures S1, S2 and S3 below show the performance of cluster solutions against the parameters outlined above. In Figure 1, two “kinks” in the WSS curve show possible optimal cluster solutions at k = 7 and k = 16 (where k is the number of clusters). While the 7-cluster solution has a lower eta-squared, it has a higher PRE than the 16-cluster solution. The Calinski-Harabasz and Duda-Hart stopping rules both show better performance for the 16-cluster solution (Figures S2 and S3). A similar observation can be made for the Kruskal-Wallis H-statistic (Figure S4), showing larger differences between groups in the clustering variable medians for a 16-cluster solution compared to a 7-cluster solution.

The tests above show that a 16-cluster solution performs better than a 7-cluster solution in terms of maximizing the differences between clusters while minimizing the differences within the clusters. However, further analysis showed that the 16-cluster solution produced 4 clusters which were too small to adequately represent all countries from which the data was collected. Removing these clusters from the solution caused the differences in some non-clustering variables between clusters to become insignificant. In addition, the partitioning of the 16-cluster solution was strongly driven by education level, household size and age range, evidenced by the clusters containing responses only from one education level, age range or household size, while preserving a mix of income classes and environmental preference levels. The 7-cluster solution, while still heavily driven by household size, exhibited fewer clusters strongly driven by a single age group or education level. In order to preserve a reasonable diversity of clusters and avoid a solution with multiple socio-demographically identical clusters (particularly as the environmental preference aggregate variations between clusters are also relatively small compared to other differences), the 7-cluster solution was ultimately selected as the best solution for this analysis.

#### Regression analysis

As the clusters were strongly differentiated by dwelling efficiency and the relative efficiency gap, a regression analysis was conducted to evaluate the effect of socio-demographic indicators, energy-saving habits and environmental preferences on these two variables (variables that were not included in the cluster analysis itself). Although none of the models could validate a hypothesis that these variables were affected by any of the tested predictors (due to the non-normal distribution of regression residuals), we briefly describe some key patterns based on the coefficients of the predictor variables, which are unbiased despite the non-validation of hypotheses and can be used to trace the relative contribution of predictors. In ordered logit and probit regressions, increasing the predictor variable by 1 unit (i.e. moving between different consecutive levels) results in an increase of ∗coefficient∗ in the log or probit odds likelihood of being in a higher efficiency gap category. A negative coefficient will mean a decrease in the likelihood of being in a higher efficiency gap category. Therefore, if increasing income from level 3 to level 4 results in a coefficient of -1.47 with p < 0.05, this means that by moving from level 3 to level 4 of income, there will be a 1.47x decrease in the likelihood of being in a higher efficiency gap category. The sign of the coefficient determines the effect of increasing the predictor on the response variable (in this case, increasing the predictor decreases the response variable) and the size of it relative to the other significant coefficients determines the size of the change in response variable when increasing the predictor (e.g. if the coefficients get smaller, it means that the effect of increasing the predictor on the response variable gets smaller).

The regression models showed that higher income, larger households and older age increased the likelihood of living in an inefficient dwelling, at population level. At cluster level, the well-off potentially efficient group was affected only by income (p = 0.015) and the resource-constrained potentially efficient only by household size (p = 0.009) and by income at p < 0.1 (p = 0.056). Note that the other 3 clusters could not be fitted with regression models due to having only one level of the dependent variable (exclusively efficient or exclusively inefficient dwellings). Like the population-level regression, higher income and larger households predicted a higher likelihood of living in inefficient dwellings, in these two clusters.

At population level, only household size had a significant effect on the relative efficiency gap: larger households had an increased likelihood of having small relative efficiency gaps, apart from the largest household group (p < 0.05), and thus a lower probability of being either very inefficient or very efficient. At cluster level, income played a significant role: in the inefficient high consumers group, wealthier households are less inefficient (p < 0.05); the same applies for the inefficient low consumers group, but only above €3,500/month, and the effect decreases with increasing income. In the resource-constrained group, both higher-income and larger households (apart from the largest) have smaller relative efficiency gaps. Given that most of the dwellings in this group are efficient, this means that in the majority of cases, higher-income and larger households are less efficient.

#### Residential buildings simulation module

A detailed outline of the core modelling concept is provided in Sachs et al. (2019), however here we provide a brief overview and clarify main concepts. The model can be summarized by four main steps. In the first step, the end-use demand of each agent is determined. Dependent on the definition of each agent, a set of possible technologies for the agent to choose from, the so-called search space, is created. Technology choice for each agent is then calculated based on the agent-specific objectives and the defined decision strategy. The last step is given by an update of the agent technology stock. Similar to (Wittmann et al., 2006) the algorithm uses different search spaces, decision strategies, and objectives for each agent to produce realistic macroscopic sectoral results.

The core of the methodology is the definition of different heterogenous agents rather than treating them as a single entity that follows a single decision method. In the presented agent- based modelling approach, each investor group is presented by an agent in the market based on the fundamental principles of bounded rationality theory (Kahneman 2003).

Each agent is defined by its own goal, attributes and methodology to solve a specific problem

(Equation 1) A={Obj,SR,DS,TP,B,MT,TS,TO,SL,PP}

including one or multiple objectives Obj, search rule SR, decision strategy DS, new or retrofit type TP, budget B, maturity threshold MT, technology stock TS, technology ownership TO, service level SL and share of the population PP.

#### Goal/objective

The investment goals are defined by different metrics, called objectives Obj, which can be grouped into economic, environmental, technology dependent aspects and personal factors. The choice of objectives can be associated with different investment planning strategies: Short-term planning, long-term planning, energy-saving, environment-friendly, and comfort-driven. Most investment makers do not fall just within one category but follow goals that combine several factors.

#### Search rules

The search rules present the first step of the decision process, the collection of information about available technologies and processing abilities of the decision makers which yield the search space of an agent. The search space presents a subset of all defined possible technologies in the residential sector. It can differ in size and can contain at maximum all possible technologies. The search space of each agent is changing over time depending on the maturity of each technology, their cost, the current technology in use, their efficiencies, and their emission characteristics. Four different categories of search rules are defined: same type, same input fuel, conventional, and all technologies for the considered end-use.

In addition to characteristic search rule of individual agents, the model aims to imitate the effect of experience with a certain technology and the observation of the maturity and integration of a technology into the market with the use of the maturity-threshold. This enables for example the model to capture time lags for adapting new technologies.

#### Decision strategies

The set of possible energy technologies that are technically and financially feasible for each agent are limited by behavioral constraints (Berger 2001) within the decision making process. The individual investment decision making among available technology and fuel alternatives is represented by different decision strategies to capture single-objective and multi-objective methods.

When the decision strategy includes multiple objectives, the approach can be based on weighted sum, epsilon constraint or a lexicography strategy, according to (Equation 2), (Equation 3), (Equation 4), as reported by Sachs et al. (2019). The weighted sum strategy transforms the set of objectives $obj_{i}$ into a single objective $obj_{WS}$ by multiplying each normalised objective $obj_{i,nom}$ by a weight $w_{i}$ according to Equation (2).

Another method for the reformulation of the objective function is given by the epsilon constraint strategy. The investment decision is carried out based on one specific objective $obj_{EC}$ while all the other objectives ($obj_{2},obj_{3}$) are constraint by some values ($\varepsilon_{2},\varepsilon_{3}$) and can be formulated according to Equation (3). All technologies that have a good performance regarding one objective but show a poor performance for the other criteria are eliminated and therefore do not belong anymore to the search space.

The third strategy is the lexicographic method, which assumes that the objectives can be ranked in the order of importance. According to the defined order of objectives, all technologies which show a good performance for a first criteria are selected. In a second step, the set of technologies is further limited by considering the second objective. The formulation of the second iteration is therefore given by Equation (4) with the optimal value of the first objective $obj_{1}^{\text{∗}}$ and a defined confidence interval $\delta_{1}$. This procedure is recursively repeated until only one technology remains. The number of technologies selected in each step depends on the defined confidence interval around the best objective.

(Equation 2) $$\text{Weighted sum }obj_{WS}=min\overset{3}{\underset{i=1}{\sum }}w_{i}obj_{i,nom}$$

(Equation 3) $$\begin{array}{l} \;obj_{EC}=\min obj_{1}\\ \text{Epsilon}-\text{constraint }obj_{2}\leq \varepsilon_{2}\\ \;obj_{3}\leq \varepsilon_{3} \end{array}$$

(Equation 4) $$\begin{array}{l} \;obj_{LEX}=\min obj_{2}\\ \text{Lexicographic }obj_{1}\leq obj_{1}^{\text{∗}}\delta_{1} \end{array}$$

#### Technology stock

Each agent owns a set of technologies, referred to as technology stock TS, which they subsequently update following the decision-making process introduced earlier. The initialization of the technology stock of each agent is carried out using additional agent attributes such as the agent type TP, technology ownership TO, and the percentage of the population that agent represents PP. An overview of all heating and lighting technologies can be found in Table S8.

#### Testing of model results

The differences between the heating and lighting technology projections of the Cluster based and top-down original approach model are statistically tested. We apply a linear model to the differences in paired data to control for nuisance parameters of the projections of both heating and lighting. We analyse significance of time and technology use differences through an ANOVA. The results show that the residuals do not follow a normal distribution, and due to the low numerosity of data points we cannot claim to be in “asymptotic territory” (see Figures S8 and S9). Therefore, the non-parametric Kruskal-Wallis is used (see Table S6), which show Technology and Technology and time to be significantly different between the cluster based and the top-down model for respectively lighting and heating projections (p < 0.001).

## Data Availability

The dataset “Categorical variables based on cross country household survey on energy consumption” that contains the aggregated cluster results data is publicly available through the zenodo platform as of the date of publication. Accession numbers are listed in the key resources table. All original code used for the cluster analysis and the the RSBM code is available for academic purposes upon reasonable request.

## Footnotes

Supplemental information can be found online at https://doi.org/10.1016/j.isci.2022.103905.
