# Supplementary material for: Translating observed household energy behavior to agent-based technology choices in an integrated modeling framework
Source: iScience. 2022 Feb 11;25(3):103905. doi: 10.1016/j.isci.2022.103905 (PMC8891977; doi:10.1016/j.isci.2022.103905)
Supplement: Document S1. Figures S1–S11 and Tables S1–S8 [file mmc1.pdf]

iScience, Volume *25*

Supplemental information

Translating observed household energy behavior to agent-based technology choices in an integrated modeling framework

Oreane.Y. Edelenbosch, Luciana Miu, Julia Sachs, Adam Hawkes, and Massimo Tavoni

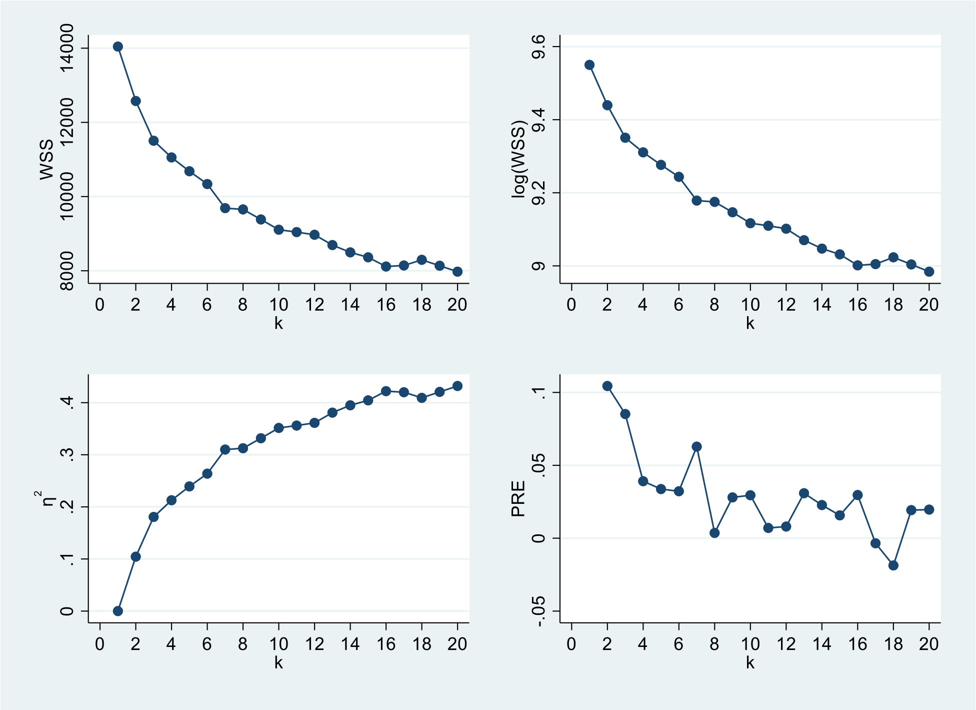

Figure S1. Visualisation of the WSS, eta-sq and PRE curves, related to Star Methods: Cluster Analysis. Results are shown for cluster solutions 1-20.

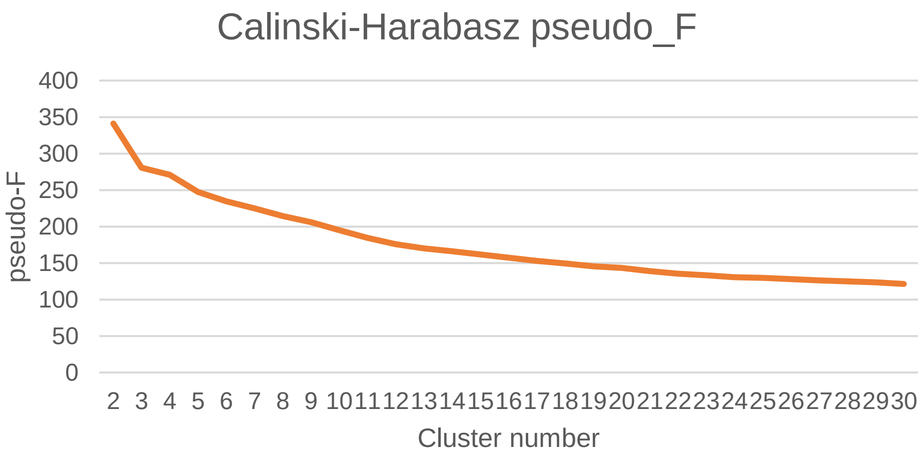

Figure S2. Calinski-Harabasz stopping rule over 2-30 cluster solutions, related to Star Methods: Cluster Analysis.

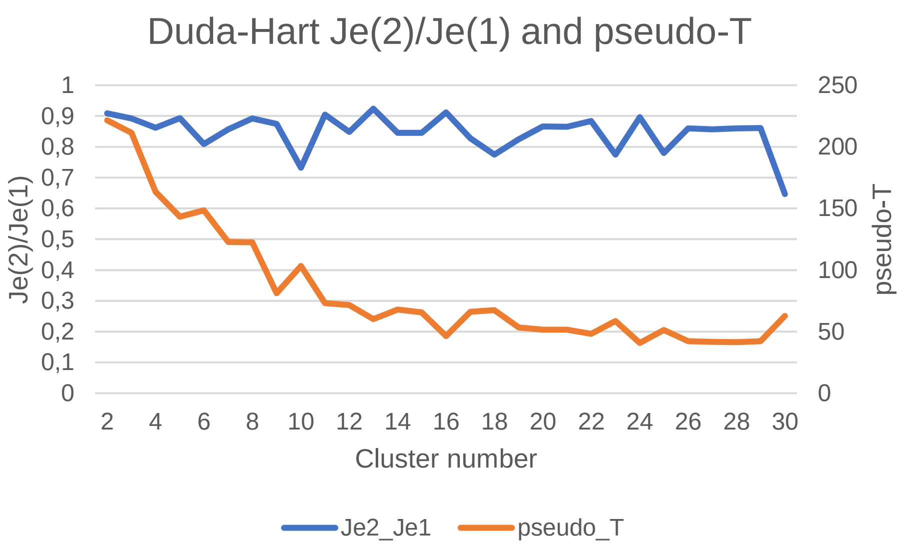

Figure S3. Duda-Hart stopping rule over 2-30 cluster solutions, related to Star Methods: Cluster Analysis.

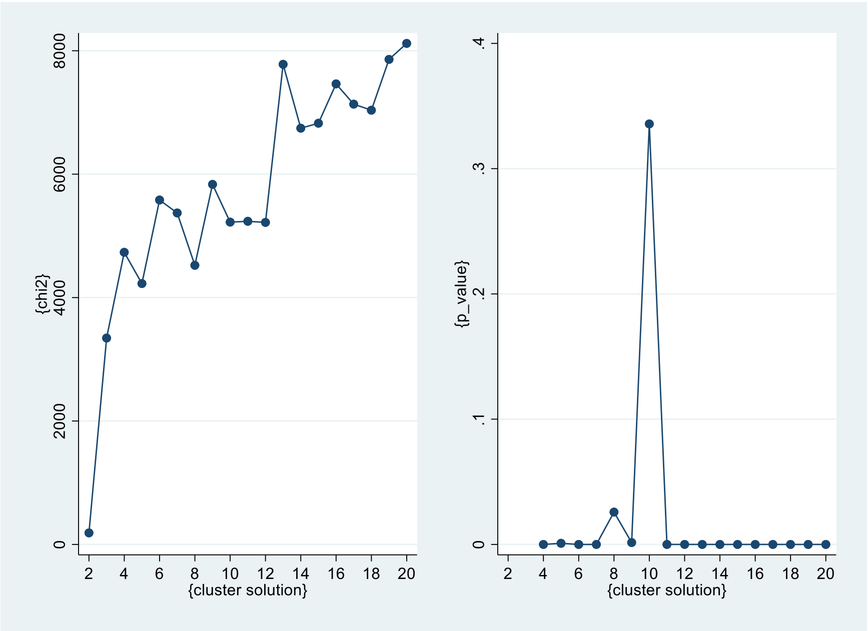

Figure S4. Kruskal-Wallis H statistic and p-value of aggregated differences between groups in clustering variables, related to Star Methods: Cluster Analysis.

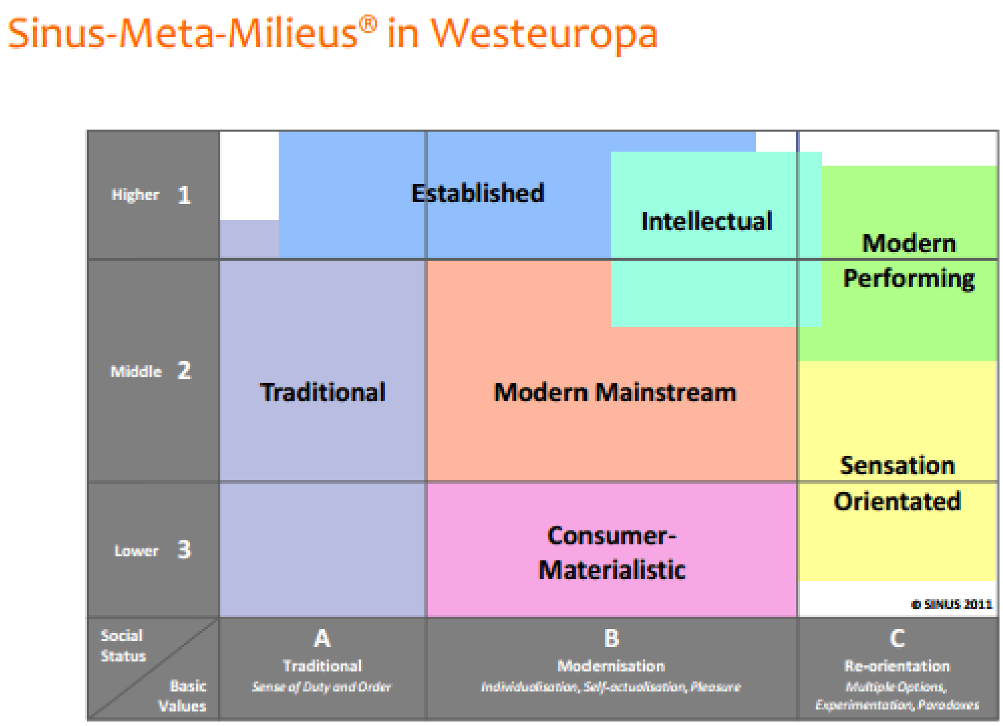

Figure S5. Seven identified population groups in Western Europe, on the basis of basic values and social status, by Sinus Milieus, related to Star Method: Method details. Traditional: The security and order loving older generation. Rooted in the world of the petty bourgeoisie or the traditional blue-collar culture; adapting where necessary; growing resignation and an ever-greater sense of being left behind. Established: The establishment in the classic sense. A responsibility and success ethic; aspirations of exclusivity and leadership along with a sense of status; growing desire for order and balance. Intellectual: The enlightened educational elite: Discerning view of the world, a fundamentally liberal outlook and post-material roots; desire for self-determination and personal development. Modern Performing: The multi-optional, efficiency-oriented performers: A global economic mindset; self-image as avant-garde when it comes to consumption and style; very technically and IT minded; establishment tendencies, erosion of visionary verve. Sensation Orientated: The fun and experience/adventure-oriented modern lower class/lower-middle class: Living in the here and now, carefree and spontaneous; often conformist at work but choosing to break free from the shackles of everyday routine in their free time. Modern Mainstream: Mainstream civil society with the will to achieve and adapt: General proponents of the social order; desire to become established at a professional and social level, seeking to lead a secure and harmonious existence; a growing sense of being out of their depth, fear and social demotion. Consumer Materialistic: The lower class in search of orientation and social inclusion ("belonging"): Desire to keep with the consumer standards of the broad middle classes - but faced with ever more social disadvantages, a sense of exclusion, embitterment and resentment.

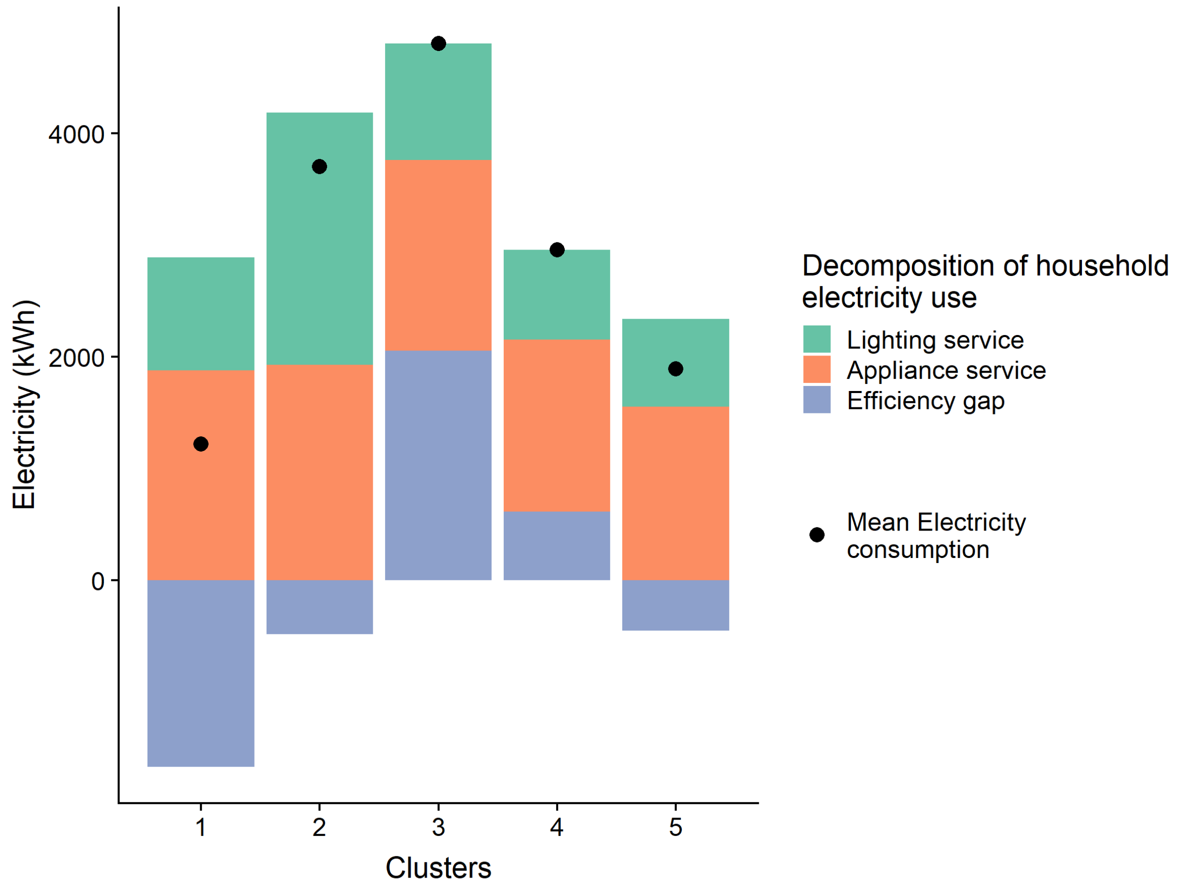

Figure S6. Annual average electricity consumption in each cluster, related to Figure 1 in the main text. The dot is the sum of the lighting service demand, appliance service demand and absolute efficiency gap equals to the average electricity consumption. T

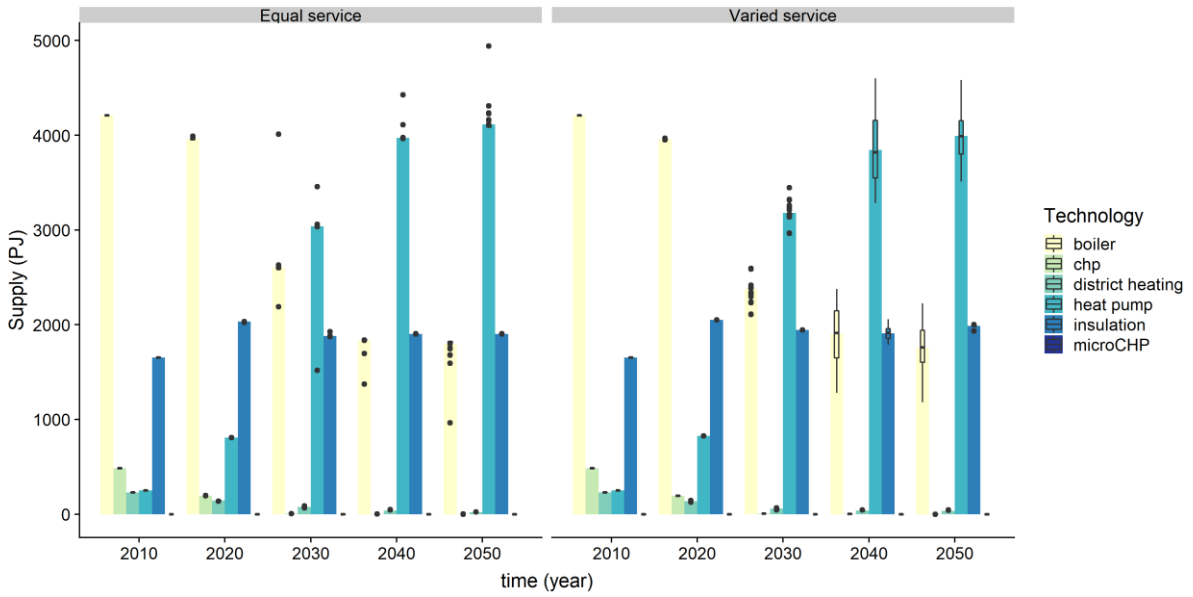

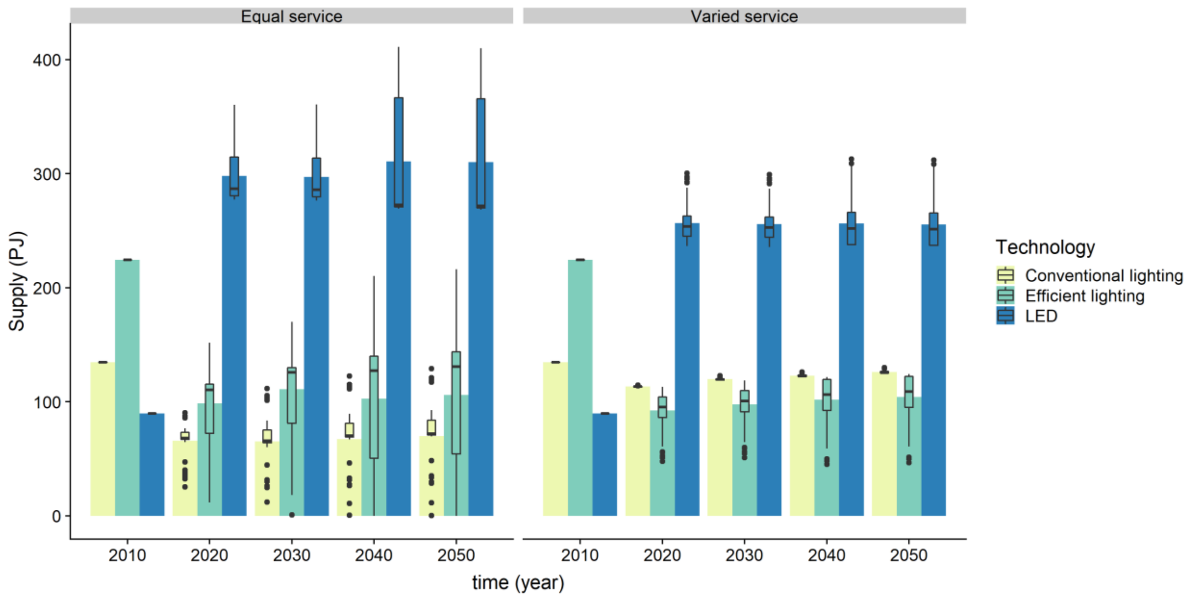

Figure S7. Heating and lighting technology supply for the equal service scenario compared to the varied service scenario, related to Figure 5 in the main text. The figure shows the mean supply across the 100 runs (indicated by the bar), and the spread in technology uptake (indicated by the boxplot). For the uptake of heating technologies, assuming equal service levels leads to an increased number of outliers but a more stable uptake trend for heat pumps and boilers, whereas incorporating varied service demand leads to more uncertainty in the uptake of these technologies towards 2050. Despite non normal residuals, given the large amount of datapoints in this case an ANOVA test can be applied. For lighting time and technologies show to be significantly different across the two models, but this is not found for the heating technologies (see Supplementary Table 7). The difference is tested through ANOVA as there are many more datapoints, therefore the asymptotic argument stands. The results in the Supplementary Table 7, show that the Technology:Scenario term, which looks for the different “differences” between the two scenarios across the technologies is significant for lighting ($p < 0.001$), while this is not the case for heating.

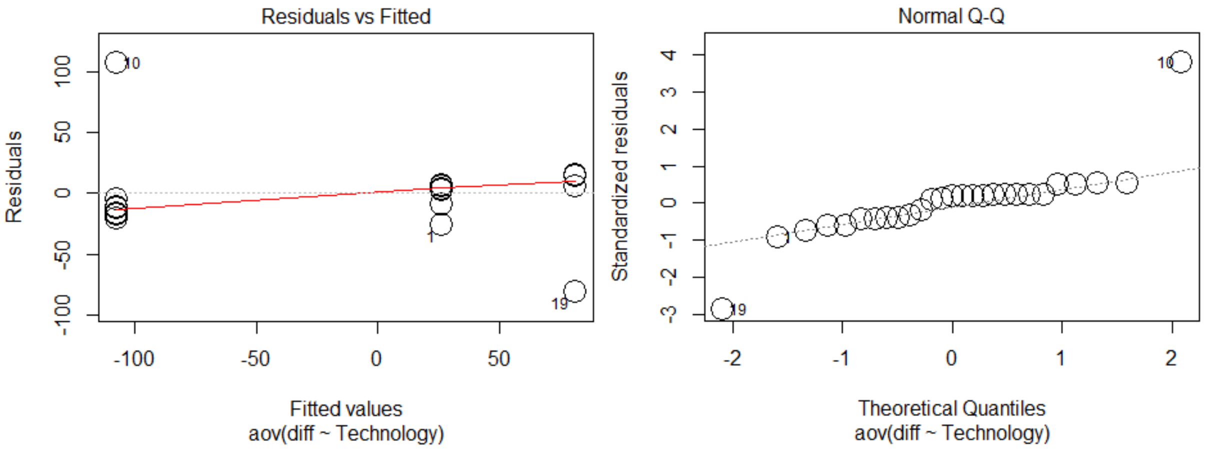

Figure S8: Lighting Heating differences between cluster-based and original runs ANOVA residuals are not normal, related to STAR methods: Testing of model results.

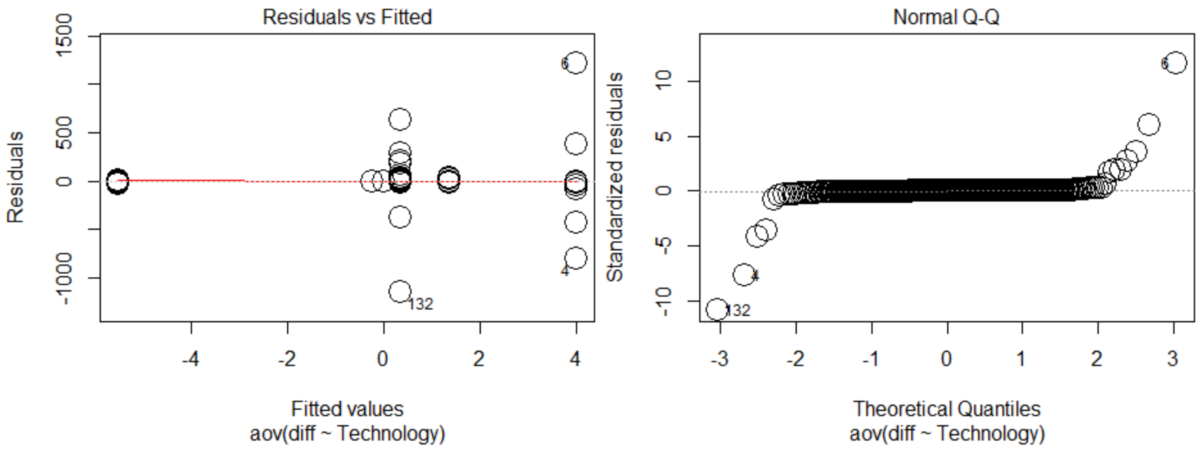

Figure S9: Heating differences between cluster-based and original runs ANOVA residuals are not normal, related to STAR methods: Testing of model results

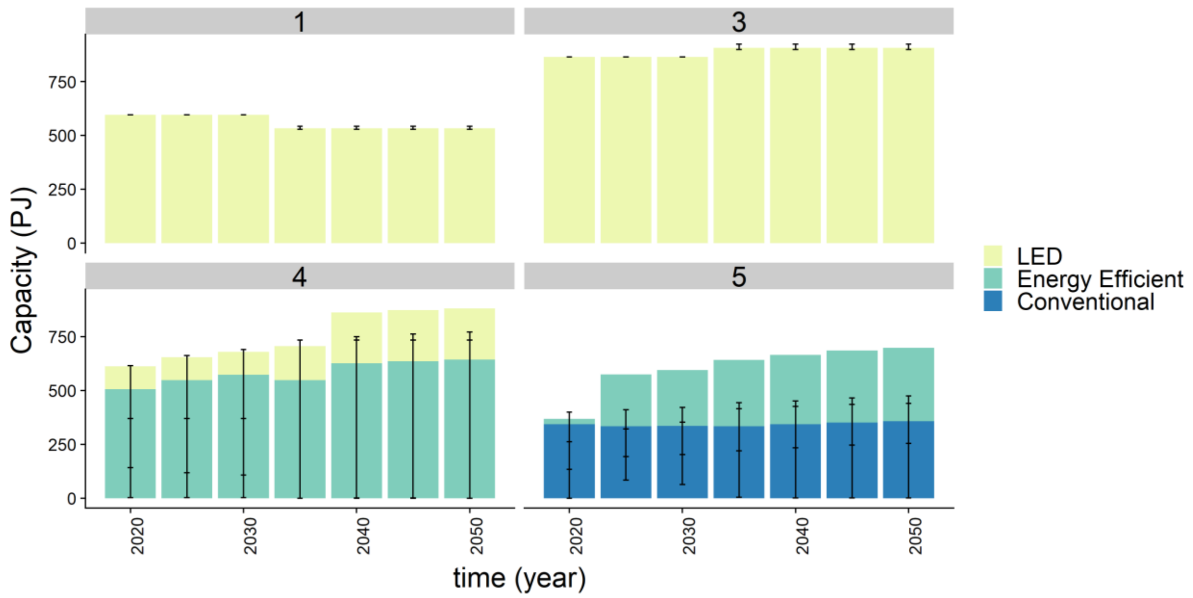

Figure S10. Lighting technology penetration in the cluster-based approach, related to Figure 6. The model is run with different service levels for the clusters 1, 3, 4 and 5 impacting the results. The figure shows the spread in technology uptake over the 100 runs, indicated by the error bar.

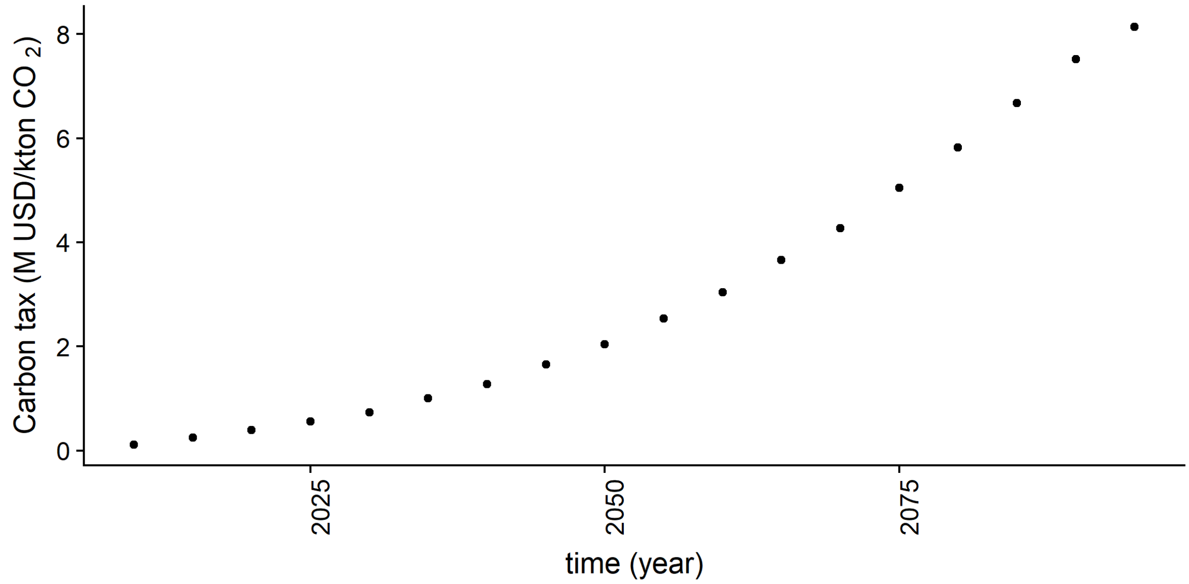

Figure S11. Carbon tax path implemented in the model runs, related to STAR Method: Method details.

Supplementary Tables

Table S1: Main variables used for the cluster analysis and secondary variables to analyse cluster characteristics, related to STAR Method: Method details.

**Main variables**	**Categories**						
income class[1]	0-1000	1000-2500	2500-3500	3500-5000	5000-7000	>7000	
age range[2]	<15	15-24	25-44	45-64	65+		
education level	primary or lower	lower secondary	vocational/ upper secondary	university (3-yr)	university (5-yr/ postgrad)		
household size	1-person	2-3 people	4-5 people	6 or more people			
environmental preference[3]	very low	low	slightly negative	average	slightly positive	high	very high
absolute efficiency gap (quintile)	very low	low	medium	high	very high		
relative efficiency gap (quintile)	very low	low	medium	high	very high		
sign of efficiency gap (dwelling efficiency)	negative (efficient)	positive (inefficient)					
lighting demand (quintile)	very low	low	medium	high	very high		
weighted appliance demand (quintile)	very low	low	medium	high	very high		
annual electricity consumption in 2016 (quintile)	very low	low	medium	high	very high		
energy-saving investments in 2016[4]	nothing	\$0-50	\$50-100	\$100-500	>\$500		
risk preference (quintiles)[5]	very risk-averse	risk-averse	neutral	risk-prone	very risk-prone		
**Secondary variables**	**Categories**						
behaviour – switching off lights	never	Rarely	sometimes	regularly	always		

[1] Income was standardized between the two datasets using publicly available tax band information and combining the income classes into sextiles.
[2] Age was standardized into ranges under UNStat guidelines.
[3] The environmental preference variable was produced by averaging each respondents' self-reported importance of environmental value, morality, identity and social approval. The averages were standardized and split into 7 quantiles.
[4] This variable was only recorded in the COBHAM survey.
[5] This variable was only recorded in the COBHAM survey, as the self-reported risk tendency of respondents (0 being risk-averse and 10 being risk-prone) There were 5 statements to respond to: "I get easily attached to material things (my car, my furniture, etc.), I would have problems with having to move to a smaller place, I tend to keep old stuff around, I feel very bad if I lose something, even when it's not that important, I think I could cope losing all my belonging in a fire, I would have no problem accepting a job that has less pay than my previous/current one.

behaviour – unplugging appliances	never	rarely	sometimes	regularly	always		
dwelling size (quintile)	<50 m^2	50-100 m^2	100-200 m^2	200-300 m^2	300-400 m^2	>400 m^2	
energy literacy[6]	low	medium	high				
home ownership	yes (owner-occupier)	no (tenant)					
share of LED lighting	less than 50%	more than 50%					
owns “luxury” electrical item?[7]	yes	no					
altruism	Continuous						
hedonism	Continuous						
value of wealth	Continuous						
length of tenure	Continuous						
environmental value[8]	Continuous						
environmental morality[9]	Continuous						
environmental identity[10]	Continuous						
environmental social approval[11]	Continuous						
bulbs per m^2	Continuous						
risk preference[9]	Continuous						

[6] Low, medium and high energy literacy are equivalent to correctly answering none, one and two, respectively, of the following questions: How much do you think it costs in terms of electricity to run: - a desktop PC for 1 hour? How much do you think it costs in terms of electricity to run: - a washing machine (load of 5kg at 60 ºC)? How much do you think is the energy saving associated with using a LED light bulb instead of a conventional Halogen bulb (with the same brightness)?
[7] Ownership of a “luxury” electrical item is defined as owning one of the following: home theatre system, sauna, solarium, swimming pool, water-bed, Jacuzzi, aquarium or terrarium.
[8] The question asked was: “Now I will briefly describe some people. Would you please indicate for each description whether that person is very much like you, like you, somewhat like you, not like you, or not at all like you ... Looking after the environment is important to this person; to care for nature and save life resources “
[9] The question asked was: “Please indicate to what extent you agree with the following statements… Acting pro-environmentally is an important part of who I am”
[10] The question asked was: Please indicate to what extent you agree with the following statements… “I feel morally obliged to save energy”
[11] The question asked was: Please indicate to what extent you agree with the following statements… “Most of the people who are important to me will approve of me when I try to save energy”

Table S2: Key characteristics of the 5 clusters, related to Figure 1, 2 and 3 of the main text.

Cluster	agent attributes						
	**education**	**age**	**household size**	**income**	**environmental preferences**	**energy literacy**	**switching lights off**
1	Vocational/Upper Secondary	45-64	2-3 people	mix	medium	medium	always
2	Vocational/Upper Secondary	45-64	2-3 people	high	medium	medium	regularly/ always
3	Vocational/Upper Secondary	45-64	2-3 people	mix	medium-high	medium	always
4	Vocational/Upper Secondary	45-64	2-3 people	mix	medium	medium	always
5	Vocational/Upper Secondary	45-64	2-3 people	low	medium	medium	Always
**Cluster**	**agent attributes**						
	**behaviour: unplugging appliances**	**lighting demand**	**weighted appliance demand**	**electricity consumption**	**absolute efficiency gap**	**relative efficiency gap**	**efficiency of dwellings**
1	mix	medium	high	low	low	high	Efficient
2	mix	high	high	high	medium-low	low	Mix
3	mix	medium	medium	high	high	medium-high	Inefficient
4	mix	low	low	medium-high	high	low	Inefficient
5	mix	low	low	low-medium	medium-low	mix	Efficient

Table S3: Overview Appliance ownership in the collected PENNY and Cobham data, related to STAR Method: Method details

Appliance type	PENNY		Cobham	
	Yes	No	Yes	No
Electric stove			119	2760
Electric oven			2623	256
Air-conditioning			1647	1232
Fridge	2556	17	2719	160
Additional fridge	611	1919	1660	1219
Freezer	1110	1430		
Dryer	975	1268	656	2223
Washing machine	2328	31	2848	31
Dishwasher	2052	520	2072	807
Add High energy consuming object	454	2120	690	2189

Table S4: Results Linear regression PENNY for each specific appliances and lighting (R) compared to sum of the appliances and lighting (L), related to STAR Method: Method details.

	Total consumption (kWh)			Total consumption (kWh)		
	Estimate	*Conf. Int.*	*p-value*	*Estimate*	*Conf. Int.*	*p-value*
(Intercept)	-851.47	-1384.78 – -318.17	.002	-1422.11	-3704.07 – 859.84	.222
Sum appliances	391.20	324.23 – 458.17	<.001			
Lighting	2.61	2.23 – 2.98	<.001	2.14	1.76 – 2.51	<.001
Fridge				1232.46	-732.36 – 3197.28	.219
Second Fridge				709.02	370.95 – 1047.10	<.001
Freezer				912.92	617.77 – 1208.07	<.001
Dryer				864.28	562.07 – 1166.48	<.001
Washing machine				389.68	-755.91 – 1535.28	.505
Dishwasher				362.98	-7.85 – 733.82	.055
Additional luxury appliance				681.02	318.14 – 1043.91	<.001
TV				22.34	-109.98 – 154.65	.741
PC				459.15	322.90 – 595.39	<.001
Observations	2024			2024		
R^2 / adj. R^2	.173 / .173			.219 / .215		

Table S5: Results Linear regression Cobham for each specific appliances and lighting (R) compared to sum of the appliances and lighting (L),), related to STAR Method: Method details.

	Total consumption (kWh)			Total consumption (kWh)		
	Estimate	*Conf. Int.*	*p-value*	*Estimate*	*Conf. Int.*	*p-value*
(Intercept)	150.22	-53.96 – 354.40	.149	231.81	-229.54 – 693.16	.325
Sum appliance	241.59	214.15 – 269.03	<.001			
Lighting	1.18	0.94 – 1.41	<.001	1.11	0.87 – 1.35	<.001
Electric stove				2.43	-210.60 – 215.45	.982
Electric oven				92.75	-57.23 – 242.74	.225
Fridge				136.76	-51.04 – 324.56	.153
Additional fridge				205.46	116.95 – 293.98	<.001
Dishwasher				164.68	65.94 – 263.42	.001
Washing machine				389.20	-21.50 – 799.91	.063
Dryer				321.84	218.78 – 424.91	<.001
Airco				235.66	149.26 – 322.06	<.001
Tvs				259.86	206.43 – 313.29	<.001
Additional luxury appliance				235.12	159.78 – 310.46	<.001
Observations	2879			2879		
R^2 / adj. R^2	.144 / .144			.159 / .156		

Table S6: Results of Kruskal-Wallis test, related to Figure 5 in main text and STAR Method: Testing of Model results.

Kruskal-Wallis	chi-squared	Df	p-value
heating diff by factor(Technology)	41.467	5	7.551e-08
heating diff by factor(time)	48.483	8	7.987e-08
lighting diff by factor(Technology)	20.568	2	3.417e-05
lighting diff by factor(time)	1.0383	8	0.998

Table S7: Results of ANOVA test, related to to Figure 5 in main text and STAR Method: Testing of Model results.

	Df	Sum sq.	Means q.	F value	Pr (>F)	Signif.
Lighting comparison						
time	1	5.67E+04	5.67E+04	15.95	6.68E-05	***
Technology	2	1.12E+07	5.60E+06	1575.08	<2.00E-16	***
Technology:Scenario	3	8.28E+05	2.76E+05	77.6	2.00E-16	***
Residuals	2993	1.06E+07	3555			
Heating comparison						
time	1	2.12E+07	2.12E+07	34.516	4.45E-09	***
Technology	5	8.52E+09	1.70E+09	2777.306	< 2e-16	***
Technology:Scenario	6	8.01E+05	1.34E+05	0.218	0.971	
Residuals	5987	3.67E+09	6.13E+05			
Signif. codes: 0 '***' 0.001 '**' 0.01 '*' 0.05 '.' 0.1 ' ' 1						

Table S8: RSBM heating and lighting technology cost, fuel and efficiency assumptions, related to STAR Method: Residential Buildings Simulation Model.

**Technology**	**Capital costs**		**Efficiency**		**Type**	**Fuel**
Year	*2010*	*2030*	*2010*	*2030*	-	-
Unit	*MUS$2010/PJ*		*%*		-	-
Boiler Biomass	40.7	35.4	0.87	0.86	boiler	biomass
Boiler Biomass_solarT	57.7	38.4	0.63	0.72	boiler	biomass
Boiler Coal	11.8	12.1	0.76	0.69	boiler	coal
Boiler Coal_solarT	17.6	15.9	0.43	0.56	boiler	coal
Boiler Electric	2.8	2.5	1.18	1.05	boiler	elec
Boiler Electriciy_solarT	8.9	10.3	1.19	1.22	boiler	elec
Boiler Hydrogen	5.2	4.3	0.71	0.93	boiler	ng
Boiler Hydrogen_solarT	10.0	10.1	1.01	0.78	boiler	ng
Boiler Kerosene	8.7	7.0	1.05	0.80	boiler	kerosene
Boiler Kerosene_solarT	12.9	13.8	1.05	0.79	boiler	kerosene
Boiler LPG	9.7	8.4	0.90	0.98	boiler	kerosene
Boiler NG	5.8	3.6	0.61	0.87	boiler	ng
Boiler NG_solarT	11.2	10.9	0.61	0.72	boiler	ng
FurnaceBiomass	36.7	33.1	0.58	0.50	boiler	biomass
FurnaceCoal	12.4	8.7	0.60	0.67	boiler	coal
FurnaceKerosene	5.7	5.8	0.55	0.59	boiler	kerosene
FurnaceLPG	6.3	5.7	0.87	0.54	boiler	kerosene

FurnaceNG	3.7	4.8	0.80	0.95	boiler	ng
CHP Biomass	32.7	20.6	0.94	0.71	chp	biomass
CHP Coal	23.5	18.9	0.70	0.57	chp	coal
CHP FuelCellHdg	317.1	187.8	0.91	0.60	chp	ng
CHP Hydrogen	26.6	19.3	0.56	0.70	chp	ng
CHP NG	15.7	20.7	0.78	0.87	chp	ng
CHP Oil	21.5		0.81		chp	kerosene
ASHeatPump	55.2	38.2	2.59	2.75	heatpump	elec
ASHeatPump_solarT	46.7	53.1	2.45	2.86	heatpump	elec
ASHeatPumpNG	15.1	15.7	3.18	2.09	heatpump	elec, ng
GSHeatPump	112.7	56.8	2.72	2.33	heatpump	elec
GSHeatPump_solarT	108.9	109.1	3.69	3.09	heatpump	elec
GSHeatPumpNG	26.3	28.8	2.59	3.11	heatpump	elec, ng
Insulation Wall Easy	85.8		1.00		insulation	heat
Insulation Wall Hard	115.4		1.00		insulation	heat
Floor Insulation	29.6		1.00		insulation	heat
Double Glazing	189.1		1.00		insulation	heat
Insulation Roof Hard	58.2		1.00		insulation	heat
Lighting Conventional	0.3	0.2	0.70	1.21	light	elec
Lighting Energy efficient	0.6	0.3	3.04	3.69	light	elec
Lighting LED	1.8	0.9	9.28	6.64	light	elec
MicroCHP Biomass	42.6	29.6	0.60	0.65	microCHP	biomass
MicroCHP Hydrogen	1497.1	90.3	0.73	1.04	microCHP	ng
MicroCHP NG	10.8	12.8	0.71	1.05	microCHP	ng
